# Identification of O-mannosylated Virulence Factors in *Ustilago maydis*

**DOI:** 10.1371/journal.ppat.1002563

**Published:** 2012-03-01

**Authors:** Alfonso Fernández-Álvarez, Miriam Marín-Menguiano, Daniel Lanver, Alberto Jiménez-Martín, Alberto Elías-Villalobos, Antonio J. Pérez-Pulido, Regine Kahmann, José I. Ibeas

**Affiliations:** 1 Centro Andaluz de Biología del Desarrollo, Universidad Pablo de Olavide, Consejo Superior de Investigaciones Científicas, Sevilla, Spain; 2 Department of Organismic Interactions, Max-Planck-Institute for Terrestrial Microbiology, Marburg, Germany; University of Melbourne, Australia

## Abstract

The O-mannosyltransferase Pmt4 has emerged as crucial for fungal virulence in the animal pathogens *Candida albicans* or *Cryptococcus neoformans* as well as in the phytopathogenic fungus *Ustilago maydis*. Pmt4 O-mannosylates specific target proteins at the Endoplasmic Reticulum. Therefore a deficient O-mannosylation of these target proteins must be responsible for the loss of pathogenicity in *pmt4* mutants. Taking advantage of the characteristics described for Pmt4 substrates in *Saccharomyces cerevisiae*, we performed a proteome-wide bioinformatic approach to identify putative Pmt4 targets in the corn smut fungus *U. maydis* and validated Pmt4-mediated glycosylation of candidate proteins by electrophoretic mobility shift assays. We found that the signalling mucin Msb2, which regulates appressorium differentiation upstream of the pathogenicity-related MAP kinase cascade, is O-mannosylated by Pmt4. The epistatic relationship of *pmt4* and *msb2* showed that both are likely to act in the same pathway. Furthermore, constitutive activation of the MAP kinase cascade restored appressorium development in *pmt4* mutants, suggesting that during the initial phase of infection the failure to O-mannosylate Msb2 is responsible for the virulence defect of *pmt4* mutants. On the other hand we demonstrate that during later stages of pathogenic development Pmt4 affects virulence independently of Msb2, probably by modifying secreted effector proteins. Pit1, a protein required for fungal spreading inside the infected leaf, was also identified as a Pmt4 target. Thus, O-mannosylation of different target proteins affects various stages of pathogenic development in *U. maydis*.

## Author Summary

The O-mannosyltransferase Pmt4 is essential for virulence of animal and plant pathogenic fungi. This protein attaches one mannose at serine/threonine residues of cell wall and secreted proteins modulating their location and function. Thus, the crucial role of Pmt4 in fungal pathogenic development is probably caused by a defective glycosylation of its target proteins altering host-fungus interaction. In this paper, we performed a screen for Pmt4 target proteins employing the fungus *Ustilago maydis*, which causes smut disease in maize plants. This allowed identifying novel Pmt4 target proteins having a crucial role on its virulence. One of these targets is the signalling mucin Msb2, a conserved protein which acts upstream of MAP kinase cascades in various fungi and regulates early pathogenic development in *U. maydis*. We propose that Pmt4-dependent glycosylation of the extracellular domain of Msb2 is required for Msb2 activity and hence pathogenic development of *U. maydis*. This is divergent to the situation in *S. cerevisiae* where the mannosylated extracellular region of Msb2p possesses a negative regulatory function. In addition, we demonstrate important roles of Pmt4 during later stages of plant infection and identified Pmt4 target proteins which could be responsible for the virulence defect of *pmt4* mutants during tumor formation.

## Introduction

O-mannosylation is an essential posttranslational protein modification in fungal cells [1]. This type of protein O-glycosylation, which adds mannoses to the nascent glycoproteins at the Endoplasmic Reticulum (ER) and Golgi Apparatus (AG), is required for correct protein conformation and stabilization [2]. In pathogenic fungi, such as *Candida albicans* or *Cryptococcus neoformans*, O-mannosylation is crucial for virulence [3]–[5].

The first step of the O-mannosylation pathway is catalyzed by protein O-manosyltransferases (Pmts), which add the first mannose to hydroxyl groups of serine and threonine in ER resident target proteins [6]. In fungi, the Pmt family is grouped into three subfamilies: Pmt1, Pmt2 and Pmt4, which mannosylate specific target proteins [7], [8]. The number of members in these subfamilies differs in each organism; for example, in *Saccharomyces cerevisiae* or *C. albicans* the Pmt1 subfamily contains two members [9], [10], while in *Schizosaccharomyces pombe*, *C. neoformans*, *Aspergillus nidulans* or in *Ustilago maydis*, the Pmt1 family contains only one member [11]–[14]. PMT orthologs have also been found in humans, rat and *Drosophila melanogaster* but not in nematodes (*Caenorhabditis elegans*) or plants (*Arabidopsis thaliana* and *Oryza sativa*) [6], [15]–[18].

Interestingly, the Pmt4 subfamily contains only one member in all organisms analyzed [6]. The protein O-mannosyltransferase Pmt4 has been characterized in several pathogenic fungi and was shown to be essential for virulence of fungal animal pathogens such as *C. albicans* and *C. neoformans* [4], [5], [10], [19]. In addition, Pmt4 is relevant for virulence in the phytopahogenic fungus *U. maydis* [11]. It has been proposed that the loss of Pmt4 in fungal pathogens causes an alteration in their ability to adhere to the host, which might be caused by affecting the glycosylation of secreted proteins and cell wall proteins [5], [20].

Despite their proposed relevance, Pmt4 target proteins have only been described in *S. cerevisiae* and *C. albicans* [21]–[23]. The lack of a defined consensus sequence that is targeted by Pmt4, coupled with the transient interaction of this O-mannosyltransferase with its target proteins, makes identification of Pmt4 substrates difficult [21]. The only knowledge regarding Pmt4 target proteins is that they should contain a membrane anchor to facilitate their glycosylation in the ER lumen. This characteristic feature has been used to perform a search for Pmt4 substrates in *S. cerevisiae* [8]. However this type of *in silico* screening has not been carried out in pathogenic fungi, which would allow the identification of fungal virulence factors. In this work, we have performed an *in silico* screening of putative Pmt4 target proteins in the plant pathogen *U. maydis* with a role during fungal pathogenesis.

*U. maydis* is a basidiomycete fungus causing smut disease in maize. For infection two yeast-like compatible haploid cells need to mate and to form a filamentous dikaryon [24]–[26]. On the leaf surface *U. maydis* senses a combination of physical-chemical plant-derived signals and differentiates into an appressorium, a morphogenetic structure which mediates fungal penetration into the plant tissues [27]. Appressorium formation and penetration is controlled by two MAP kinases, Kpp2/Ubc3 and Kpp6 [28]–[30]. Moreover, it is known that the perception of the physical surface signal required for appressorium formation depends specifically on the Kpp2/Ubc3 MAP kinase [27], and that its activation is likely to involve the signalling mucin Msb2 [31]. Msb2 has been placed upstream of the MAP kinase pathway, consisting of the MAPKK kinase Kpp4/Ubc4, the MAPK kinase Fuz7/Ubc5 and the MAP kinase Kpp2/Ubc3 [28], [30], [32]–[34]. After penetration of the plant epidermis, *U. maydis* proliferates inside the plant as a mycelium. During this process the fungus produces high amounts of putative secreted proteins which are only found in related smut fungi and are important for virulence [25], [35]. Many of the respective effector genes are organized in gene clusters [25]. At late stages of the fungal invasion process, *U. maydis* induces the formation of prominent tumors in the plant which will finally contain the diploid spores [24], [36], [37].

The Pmt4 protein is the only O-mannosyltransferase required for pathogenesis in *U. maydis*. The deletion of *pmt4* causes a drastic reduction in appressorium formation and, more importantly, a total loss of fungal capacity for plant penetration [11] (Figure S1). In this work, we present novel Pmt4 target proteins, identified by an *in silico* screening, with an essential role during *U. maydis* pathogenic development. Among them, we detect the signalling mucin Msb2 as a Pmt4 substrate, suggesting that an altered glycosylation pattern of this sensor membrane protein could be the cause for the defects in appressoria production in Δpmt4 cells.

## Results

### Pmt4 substrate screening on the *U. maydis* proteome

**Figure 1:**
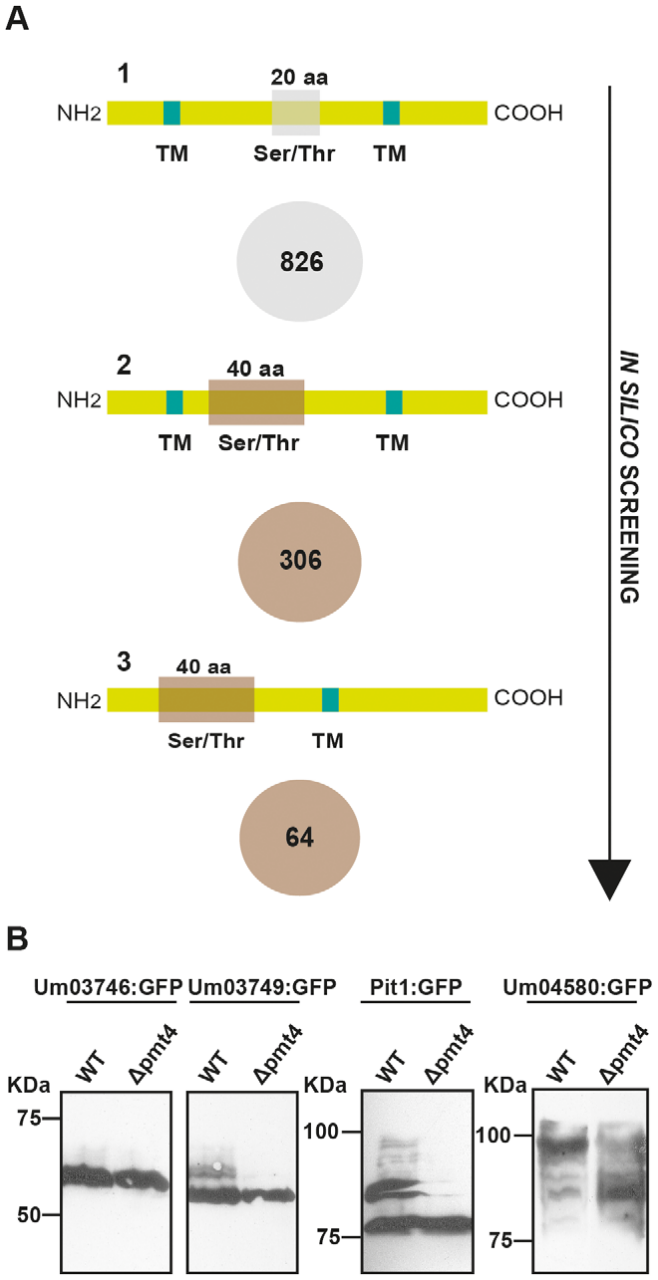
Design and validation of the *in silico* screening of Pmt4 target proteins. **A.** In the first step, we selected those proteins containing plasma membrane anchoring and a window of 20 aa where the percentage of Ser/Thr was ≥40%. 826 of the 6787 proteins contained in the *U. maydis* proteome (MIPS) fulfilled both conditions. In the second stage, we required a wider Ser/Thr rich region, of 40 aa containing a percentage of Ser/Thr ≥40%. We found 306 proteins. Finally, we searched for those containing the Ser/Thr rich region in the luminal part of the protein. With this filter, the list was reduced up to 64 putative Pmt4 target proteins. **B.** Validation of the *in silico* screening of Pmt4 target proteins in *U. maydis*. The figure shows the western blot analysis of Um03746 (∼34 KDa), Um03749 (∼30 KDa), Pit1 (∼48 KDa) and Um04580 (∼36 KDa) proteins tagged with GFP (∼27 KDa) in the SG200 (WT) and SG200Δpmt4 backgrounds. Um03749, Pit1 and Um04580 suffer a differential glycosylation processing in the *pmt4* mutant since we did not observe the bands that probably correspond to the glycosylated fraction of the proteins. The Um03746 processing does not seem to be affected by the loss of *pmt4*.

Since the role of Pmt4 in virulence must be mediated via its target proteins, we performed a search for such substrates in order to explain the role of Pmt4 in appressorium formation and penetration. Thus, we devised a bioinformatic approach to identify putative Pmt4 targets from the 6787 proteins contained in the *U. maydis* proteome. As Pmt4 mediates O-mannosylation of Ser/Thr-rich membrane-associated proteins [8], we carried out three sequential *in silico* searches. First, we identified membrane proteins containing a region of at least 20 aa in which the percentage of Ser/Thr was ≥40%. With these parameters we found 826 proteins (12.17% of the *U. maydis* proteome) (Table S1). Then, we performed a more restrictive search on the *U. maydis* proteome for membrane attached proteins containing a wider region of 40 aa, where the percentage of Ser/Thr was ≥40%. With this bioinformatic approach we identified 306 proteins from the *U. maydis* database (4.51% of the *U. maydis* proteome) (Table S2). Since it has been described that the presence of a Ser/Thr rich region in the amino terminal part of single-pass transmembrane proteins triggers the recognition by Pmt4 [8], this was the search criterion for the third round. 64 out of the 306 previously identified sequences met these criteria (0.94% of the *U. maydis* genome) (Table S3) (see Figure 1A).

The obtained putative Pmt4 substrates were categorized based on their probable functions using *FunCatDB* from the MIPS *U. maydis* database (http://mips.helmholtz-muenchen.de/genre/proj/ustilago/). We found that the initially identified group of 826 proteins was mainly enriched in proteins associated with transport, i.e. ion transport, detoxification by export as well as homeostasis of cations. The more restricted group of 306 proteins contained mostly unclassified proteins (52.1% of the proteins contained in the list). Other enriched functional categories were associated with inorganic chemical agent resistance and detoxification by export. Finally, the most restrictive group of 64 proteins was also mainly enriched in unclassified proteins (Figure S2).

In the genome of *U. maydis* many of the genes encoding small secreted proteins are organized in gene clusters, most of them implicated in virulence [25], [35]. Interestingly, we found in our screening four proteins included in these clusters: Um01235, Um03746 and Um03749 (identified in group 1, Table S1); and Um01374 (belonging to group 2, Table S2). The Um01235 protein is part of the cluster 2A. The deletion of all eight genes from cluster 2A causes a hypervirulent phenotype [25]. On the other hand, Um03746 and Um03749 reside in cluster 10A, which contains 10 genes for effector proteins in total. The deletion of cluster 10A reduces virulence [25]. Finally, Um01374 (Pit1) is one of four proteins encoded by the *pit* (proteins important for tumors) cluster. The deletion of the transmembrane gene *pit1* or the effector gene *pit2* strongly reduces pathogenic development at the level of tumor formation [38].

### *In silico* screening validation

To confirm our bioinformatic results, we performed a biochemical approach to study the role of Pmt4 in posttranslational modification of its putative target proteins by western blot. For this purpose we isolated candidate proteins from wild-type and Δpmt4 strains and compared the mobility of these proteins during SDS polyacrylamide gel electrophoresis (SDS-PAGE). Given the critical role of cluster 10A during *U. maydis* virulence, we decided to analyse the role of Pmt4 in the posttranslational modification of Um03746 and Um03749. In addition we included the protein Pit1. Finally, we also randomly selected an until now uncharacterized protein, Um04580, which contains a stretch of 40 aa where the percentage of Ser/Thr is ≥40%. The deletion of *um04580* did not affect *U. maydis* virulence (Figure S3).

Since some of the candidate genes are expressed exclusively during biotrophic development, i.e. *um03746*, *um03749* and *um01374* [25], [38], we placed the open reading frames (ORFs) of the candidate genes under the control of the constitutive active *otef* promoter [39]. A C-terminal fusion to *gfp* allowed visualization of the produced proteins by western blot. The constructs were integrated into the *ip locus* of the solopathogenic SG200 strain and its derivate SG200Δpmt4. Cells were grown in YEPSL liquid medium, total cell extracts prepared and proteins were separated by SDS-PAGE. In western blot analysis Um03749-GFP, Pit1-GFP and Um04580-GFP, but not Um03746-GFP showed differences in mobility when isolated from the two strains (Figure 1B). The higher mobility observed when *pmt4* is deleted suggests that these proteins could be direct Pmt4 targets. Thus, in analogy with *S. cerevisiae*, the bioinformatic approach is a useful tool to identify new Pmt4 target proteins.

In order to substantiate the role of protein glycosylation on the O-mannosylated virulence factors identified, we investigated if the novel Pmt4 target proteins, Pit1 and Um03749, are also N-glycoproteins. N-glycosylation consists in the addition of an oligosaccharide core to nascent protein in the consensus sequence Asn-X-Ser/Thr (where X could be any acid amino except proline). Similarly to O-mannosylation, N-glycosylation is also required for full virulence of *U. maydis* [40]. In Um03746 and Um03749 no putative glycosylation sites could be identified. In Pit1 we identified the putative N-glycosylation site NGTF (amino acids 330–333). To know if Pit1 is also modified by N-glycosylation we analysed the Pit1-GFP protein in the SG200Δcwh41 background. Cwh41 encodes glucosidase I, an upstream component of the N-glycosylation machinery in *U. maydis* [41]. Pit1-GFP isolated from Δcwh41 strains showed a similar mobility shift during SDS-PAGE as Pit1-GFP isolated from Δpmt4 strains. This observation indicates that the glycosylated isoform of Pit1 is also N-glycosylated (Figure S4).

### Msb2 is a Pmt4 substrate in *U. maydis*

To understand the specific role of *pmt4* during *U. maydis* appressorium formation and penetration we searched our list of putative Pmt4 target proteins for those for which a link to appressoria had previously been established. Interestingly, in group 3 we identified Um00480 (Msb2), a transmembrane mucin, which plays an important role during early pathogenic development in *U. maydis* [31]. Recently, homologs of Msb2 have been also linked to virulence in the plant pathogenic fungi *Magnaporthe oryzae* and *Fusarium oxysporum* [42], [43]. Msb2 meets the most restrictive criteria for Pmt4 targets by displaying a window of 40 aa with a percentage of ≥40% Ser/Thr located in the amino terminal part of the protein (Table S3). Furthermore, *S. cerevisiae* Msb2p has been shown to be glycosylated by Pmt4p [44]. Although *U. maydis* Msb2 is unable to functionally complement the *MSB2* mutant of *S. cerevisiae* and displays only weak similarity to *S. cerevisiae* Msb2p [31], we took this as a strong indication that Pmt4 glycosylates Msb2 in *U. maydis*.

**Figure 2:**
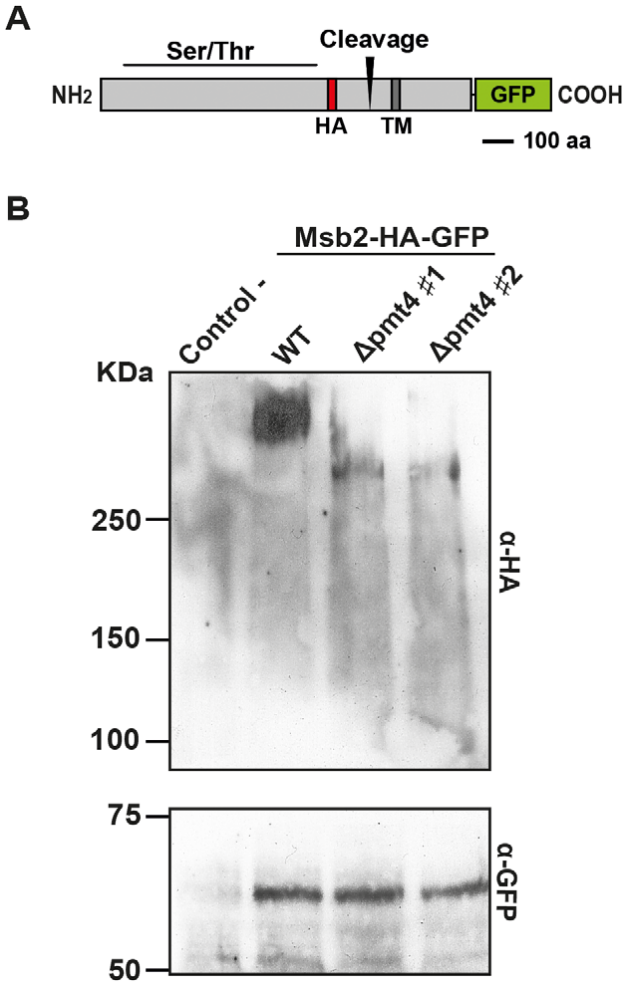
Msb2 is a Pmt4 substrate in *U. maydis*. **A.** Domain architecture of the Msb2-HA-GFP fusion protein. The HA-epitope is integrated at amino acid 709 and the C-terminus is fused to GFP. The Ser/Thr rich region as well as the predicted proteolytic cleavage site is indicated. **B.** Western Blot analysis of Msb2-HA-GFP isolated from SG200Δmsb2/msb2-HA-GFP (WT) and two independent clones of SG200Δmsb2Δpmt4/msb2-HA-GFP (#1 and #2, respectively). SG200 was used as a control. The first gel (above) contains 6% of polyacrylamide and α-HA antibody was used to detect the N-terminal part of Msb2. The other gel (below) contains 10% of polyacrylamide and was treated with α-GFP antibody to detect the C-terminus of Msb2. Equal amounts of proteins of total cell extracts were loaded in each lane.

In *S. cerevisiae*, Msb2p is cleaved upstream of the transmembrane domain releasing an extracellular N-terminal part and a cellular C-terminal fragment [45]. To analyse the Msb2 protein in *U. maydis* we used a differentially tagged protein, which was C-terminal fused to GFP and carried an internal HA-epitope between amino acids 709 and 710 in the extracellular region (Figure 2A). The *msb2-HA-GFP* construct was placed under the control of the *otef* promoter and integrated into the *ip locus* of SG200Δmsb2. Western blot analysis revealed that the Msb2 protein is processed into two distinct fragments. The extracellular domain, detected by the anti-HA-antibody, migrated at a molecular weight of >250 kDa, while the anti-GFP-antibody detected a product of approximately 65 kDa. The size of the latter fragment allowed us to predict a cleavage site, situated between the Ser/Thr rich region and the transmembrane domain (Figure 2). In *S. cerevisae*, cleavage of Msb2 results in the secretion of the N-terminal extracellular domain [45]. Similar to the situation in *S. cerevisiae* we could detect the extracellular N-terminal domain of *U. maydis* Msb2 in the culture supernatant, while the C-terminal fragment was exclusively detected in the cellular fraction (Figure S5).

To confirm that Msb2 is a Pmt4 target protein in *U. maydis*, we deleted *pmt4* in SG200Δmsb2/msb2-HA-GFP and analysed whether *pmt4* deletion influences the migration of the Msb2 protein in SDS-PAGE. The extracellular part of Msb2 isolated from Δpmt4 strains migrated faster in polyacrylamide gel electrophoresis than the extracellular part of Msb2 isolated from wild-type strains (Figure 2B). Thus, we can conclude that Msb2 is a mannosylated substrate of Pmt4 and that mannosylation occurs in the extracellular domain of Msb2. However, the extracellular fragments from wild-type and Δpmt4 strains migrated at positions >250 kDa, which exceeded by far the predicted size of the full length Msb2 protein of ∼145 kDa. This indicates that Msb2 is also postranslationally modified by Pmt4-independent mechanisms. We analysed the Msb2 protein in the N-glycosylation deficient Δcwh41 strain. We found that in SDS-PAGE the extracellular part of Msb2 migrated similarly in both, wild-type and Δcwh41 background (Figure S4). Thus, our data suggest that, similarly to the situation described in budding yeast [44], Msb2 in *U. maydis* is subject to a non-characterized posttranslational modification process.

### The Ser/Thr rich region of Msb2 is required for *U. maydis* virulence

**Figure 3:**
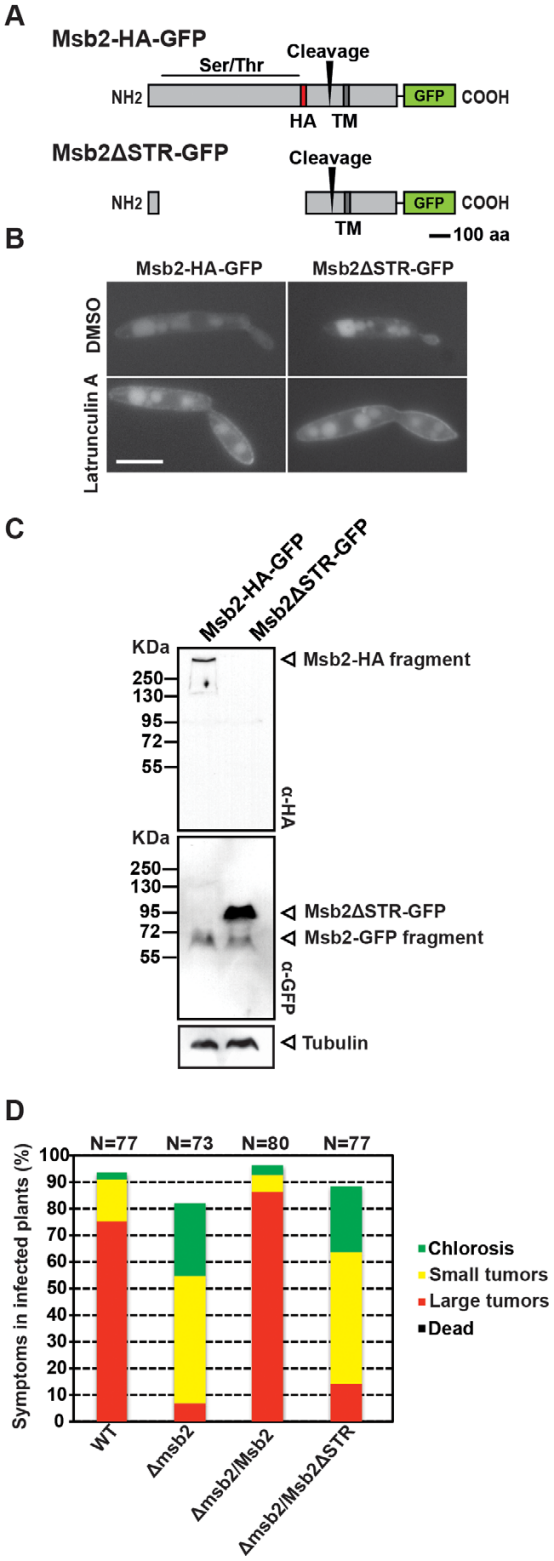
The STR region is required for Msb2 function in *U. maydis*. **A.** Schematic model of the protein variant Msb2ΔSTR-GFP compared to Msb2-HA-GFP. The deletion of the coding region of the Ser/Thr rich domain of Msb2 is shown. **B.** Cells of SG200Δmsb2/msb2-HA-GFP and SG200Δmsb2/msb2ΔSTR-GFP were grown to mid log phase in YEPSL and analyzed microscopically without addition (top row) or after addition of latrunculin A (bottom row). The fluorescent signal corresponding to GFP is shown. **C.** Total protein extracts of the indicated strains were subjected to western analysis with α-HA antibody (upper panel) and α-GFP antibody (middle panel). Tubulin served as loading control and was detected with α-tubulin antibody (bottom panel). The fusion proteins and processed fragments are indicated by arrowheads on the right. The molecular weight ruler is depicted on the left. **D.** Disease symptoms caused by SG200 (WT), SG200Δmsb2, and SG200Δmsb2 complemented with either Potef:msb2 or Potef:msb2ΔSTR were scored 12 dpi. *N* indicates the total number of plants evaluated in each case.

Considering that the extracellular domain of Msb2 is mannosylated by Pmt4, we assumed that this domain might be needed for the function of Msb2. We therefore deleted the coding region of the Ser/Thr rich domain of Msb2 and integrated the construct into the *ip locus* of SG200Δmsb2 generating the variant SG200Δmsb2/msb2ΔSTR-GFP (Figure 3A). The loss of this region did not alter the normal localization of Msb2 in the plasma membrane as revealed by treatment with the endocytosis inhibitor Latrunculin A [31] (Figure 3B). Western blot analysis revealed that processing of the truncated Msb2 protein still occurred since the C-terminal 65 kDa fragment could be detected for both full length Msb2-HA-GFP and truncated Msb2ΔSTR-GFP (Figure 3C). However, in plant infections no complementation was observed when the truncated Msb2 protein was expressed, while virulence could be completely restored when Msb2 was expressed as full-length protein (Figure 3D). This indicates that, contrary to the situation in *S. cerevisiae*, where the Ser/Thr rich region of Msb2p possesses a negative regulatory function [46], the mannosylated Ser/Thr-rich region of Msb2 in *U. maydis* has a positive role during virulence.

### Genetic interaction analysis between *pmt4* and *msb2* suggests that both regulate appressorium differentiation by the same pathway

In *U. maydis*, appressorium formation requires a set of combined physical-chemical plant-derived signals [27]. Δmsb2 strains are capable of recognizing the chemical signal (hydroxy fatty acids) but show defects in responding to the physical stimulus (hydrophobic surface). Hence, the *msb2* mutant is severely affected in appressorium differentiation [31]. To investigate whether the defect in appressorium formation described for the *pmt4* mutant might be due to a defective sensing activity of Msb2, we analysed the capability of Δpmt4 cells, as well as the double mutant Δpmt4Δmsb2 to respond to physical-chemical signals.

**Figure 4:**
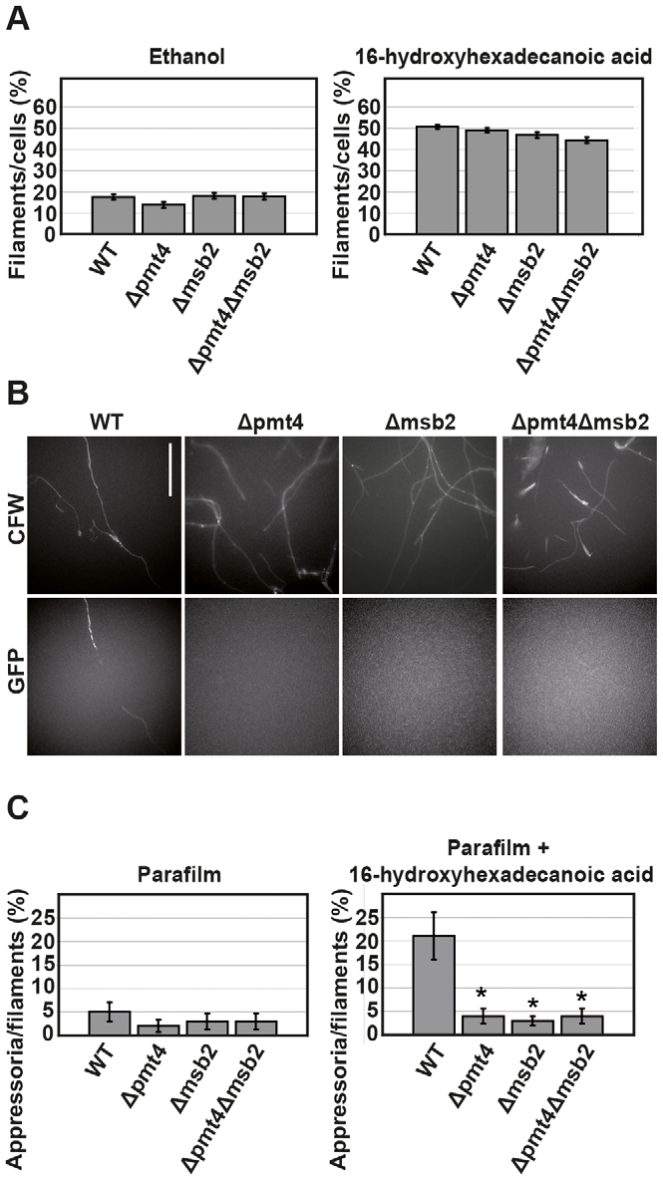
The *pmt4* and the double *msb2 pmt4* mutants respond to hydroxy fatty acid but not to hydrophobic surfaces. **A.** Chemical plant-derived signals reception does not depend on Pmt4. SG200 (WT) and its derivative mutants were grown to exponential phase and incubated in liquid medium (2% YEPSL in water) supplemented with 100 µM of 16-hydroxyhexadecanoic acid dissolved in ethanol or ethanol (control) for 18 hours at 28°C. Filament formation was analyzed. All data are shown as mean values ±SEM of three independent repetitions quantifying 200 cells in each case. **B.** Physical signals reception depends on Pmt4. SG200AM1 (WT) and its derivative mutants were sprayed as cell suspension (2% YEPSL) on parafilm M with or without 100 µM 16-hydroxyhexadecanoic acid and incubated for 18 hours at 28°C. Cells were stained with calcofluor white (CFW) and appressorium formation was scored visualizing the expression of the GFP reporter gene. Appressorium formation was reduced in all analysed mutants. Scale bar represents 50 µm. **C.** Quantification of appressoria production. The assay was performed as described in B. In three independent experiments, approximately 100 filaments per strain and experiment were analyzed with respect to appressorium formation. All data are shown as mean values ±SEM. Asterisk indicates statistically significant differences between wild-type (control) and each mutant. Single asterisk means P value≤0.001.

To test the chemical signal perception, we used 16-hydroxy hexadecanoic acid, a main component of the maize cuticle. Exposure of SG200 to this fatty acid induces filamentous growth [27]. Filament formation of SG200Δpmt4, SG200Δmsb2 and SG200Δmsb2Δpmt4 cells in response to 16-hydroxy hexadecanoic acid was indistinguishable from SG200 cells (Figure 4A). Thus, the defect in appressorium formation described for Δpmt4 cells is not likely to be linked to a defective perception of the chemical signal.

To study the perception of the physical signal, we added 16-hydroxy hexadecanoic acid to *U. maydis* cell suspensions and sprayed the cells onto parafilm M, which constitutes a hydrophobic and hard surface inducing differentiation of hyphae into appressoria [27]. To facilitate quantification of appressoria, we used strains harbouring a *gfp* reporter, AM1, which is specifically expressed in those hyphae that have formed an appressorium [27]. We observed comparable filament formation of SG200AM1 and the respective derivative mutants. However, with respect to appressorium formation, *msb2*, *pmt4* and the double *msb2 pmt4* mutants were severely reduced (Figure 4B). Interestingly, appressorium formation in the various mutants was impaired to a similar extent (Figure 4C) suggesting that the defect in appressorium development in the Δpmt4 strain might be a consequence of a defect in glycosylation of the Msb2 protein.

### Pmt4 and Msb2 are likely upstream components of the MAP kinase pathway

**Figure 5:**
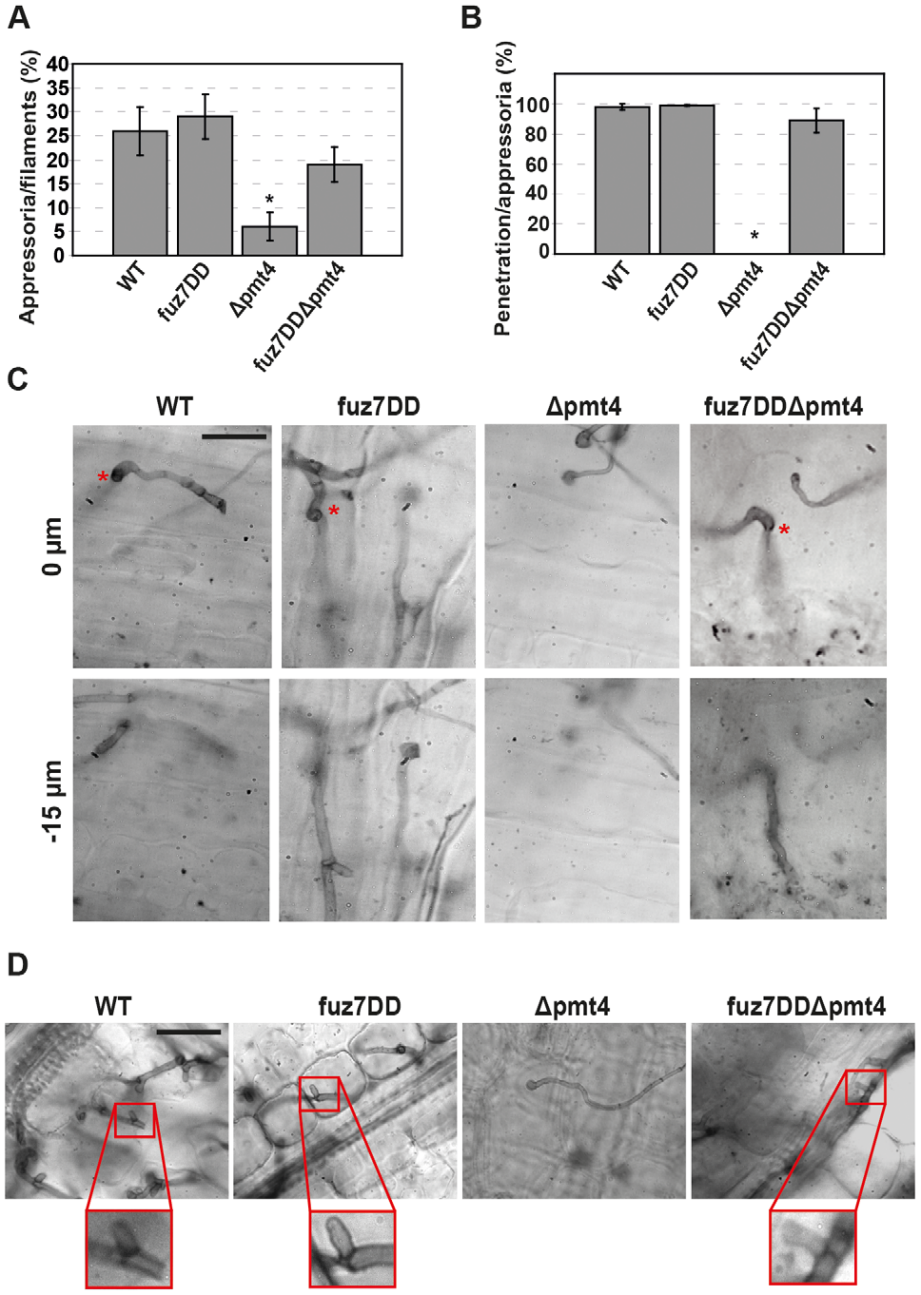
The hyperactive allele *fuz7DD* rescues the appressorium formation and penetration defects of the Δpmt4 strain. **A.** Seven day old maize seedlings were infected with the strains SG200 (WT), SG200fuz7DD, SG200Δpmt4 and SG200fuz7DDΔpmt4. Appressoria production was scored 15 hours later quantifying >100 filaments in each case. Data are shown as mean values ±SEM. Asterisk indicates statistically significant differences between wild-type (control) and Δpmt4 strains, P value≤0.001. **B.** Plant penetration was evaluated quantifying appressoria penetration versus appressoria on plant leaves stained with chlorazol black two days after infection. The quantification was performed with the strains indicated. Data represent the mean values ±SEM. The number of appressoria analysed in each case was ≥50. Asterisk indicates statistically significant differences between wild-type (control) and Δpmt4 strains (P value≤0.001). **C.** Appressorium penetration is normal in the SG200fuz7DDΔpmt4 strain. The *z* axis image projections show the site of penetration (0 µm) and the hyphae invading into plant cells (−15 µm). Δpmt4 strain is the only one unable to penetrate the plant cuticle. **D.** Infected leaves were stained with chlorazol black two days after inoculation. Hyphae of the solopathogenic strains SG200 (WT), SG200fuz7DD and SG200fuz7DDΔpmt4 form clamp-like structures *in planta* (framed in red). However clamp-like structures are not observed in SG200Δpmt4 infections only recognize appressorium formation on the plant surface. Bar represents 20 µm.

**Figure 6:**
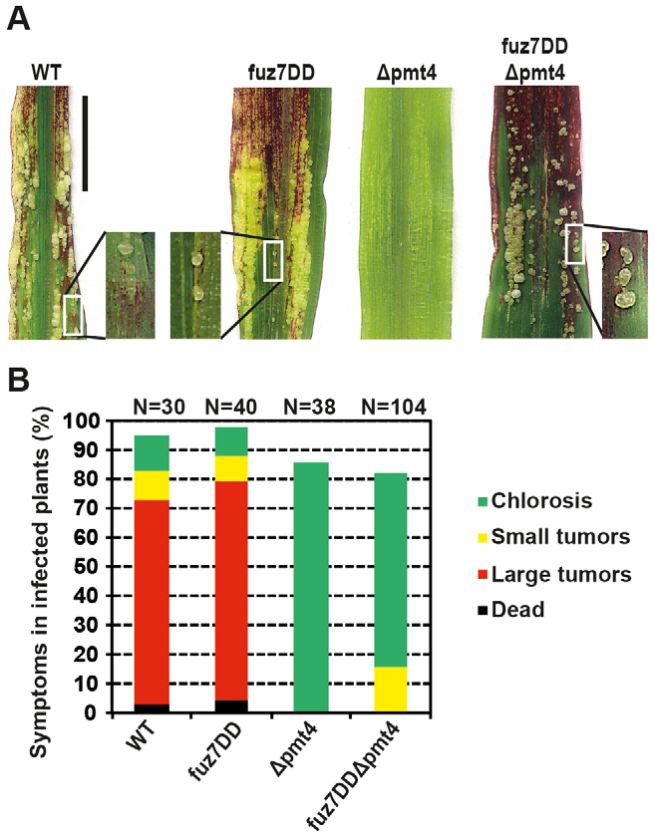
Expression of *fuz7DD* in the Δpmt4 strain partially restores the tumor induction in maize. **A.** Disease progression in plant infections with the strains indicated. No infection symptoms were detected on plants inoculated with the SG200Δpmt4 strain. The SG200fuz7DDΔpmt4 induces anthocyanin production and small tumors formation. Bar represents 2 cm. **B.** Plants were infected with the indicated strains and symptoms were scored 12 days post-infection. *N* indicates the total number of plants evaluated in each case.

In *U. maydis* Msb2 has been placed upstream of the MAP kinases Kpp2 and Kpp6 implicated in appressorium development and penetration respectively [27], [29], [31], [34]. In fact, expression of a hyperactive allele of the MAP kinase kinase Fuz7, Fuz7DD [34], which activates Kpp2 and Kpp6, restores plant cuticle penetration in *sho1 msb2* double deletion strains [31], while the *sho1 msb2* double deletion strain is non-pathogenic and unable to form appressoria on the plant surface [31]. The genetic interaction analysis between *msb2* and *pmt4* may suggest a role of *pmt4* upstream of the MAP kinase cascade. However, we cannot exclude the possibility that Pmt4 is acting downstream of the MAP kinase pathway controlling glycosylation of proteins implicated in the appressorium morphogenesis. To test our hypothesis that Pmt4 acts above the MAP kinase cascade we used the hyperactive allele *fuz7DD* cloned under the control of the *crg* promoter, which is repressed by glucose and induced by arabinose [31], [47]. This construct was integrated in the *ip locus* of SG200, SG200Δpmt4 and SG200Δmsb2. To analyse appressorium formation, we infected leaves from seven days old maize seedlings with SG200, SG200fuz7DD, SG200Δpmt4, SG200fuz7DDΔpmt4, SG200Δmsb2 and SG200fuz7DDΔmsb2 in the presence of arabinose. To quantify appressorium formation, cells were stained with calcofluor white 15 hours after plant inoculation. Induction of the *fuz7DD* allele restored appressoria production in the *pmt4* and *msb2* mutants (Figure 5A and Figure S6A). We also analysed the appressorium-mediated penetration under these conditions. To this end, we used the chlorazol black E staining procedure to visualize invading hyphae [29]. Remarkably, the appressorium-mediated penetration capability of Δpmt4 cells was also restored upon expression of the constitutively active *fuz7DD* allele, including the formation of clamp-like cells, the structures associated with fungal progression inside the plant [48] (Figure 5B, 5C and 5D). Analysis of disease progression 12 days after infection revealed an increased virulence capacity of SG200fuz7DDΔpmt4 and SG200fuz7DDΔmsb2 when compared to SG200Δpmt4 and SG200Δmsb2, respectively (Figure 6 and Figure S6B). Thus, our data suggest that the role of Pmt4 on appressorium biology might take place upstream of MAP kinase cascade.

If Pmt4 and Msb2 activate the MAP kinase cascade during appressorium formation, Kpp2 phosphorylation in the respective deletion mutants is expected to be reduced during this developmental stage. To examine the phosphorylation of Kpp2 we incubated SG200, SG200Δpmt4 and SG200Δmsb2 for 10 h on parafilm M. At this time point about 15% of the SG200 filaments begin to form appressoria (data not shown). As shown in Figure S7, the hydrophobic surface induced Kpp2 phosphorylation in SG200 as well as in the mutants. We observed a slight reduction in Kpp2 phosphorylation in the Δpmt4 (84±11%) and Δmsb2 (76±7%) strains when compared to SG200 (100%) (Figure S7). However, compared to SG200 only the Δmsb2 strain showed a statistically significant reduction in Kpp2 phosphorylation (P<0.05), while differences in the Δpmt4 strain were not statistically significant (P>0.05; Figure S7). This experiment shows that both, Pmt4 and Msb2, are marginally involved in the activation of the MAP kinase Kpp2.

### Scanning Electron Microscopy suggests that the deletion of *pmt4* causes post-appressorial outgrowth of hyphae due to a penetration defect

**Figure 7:**
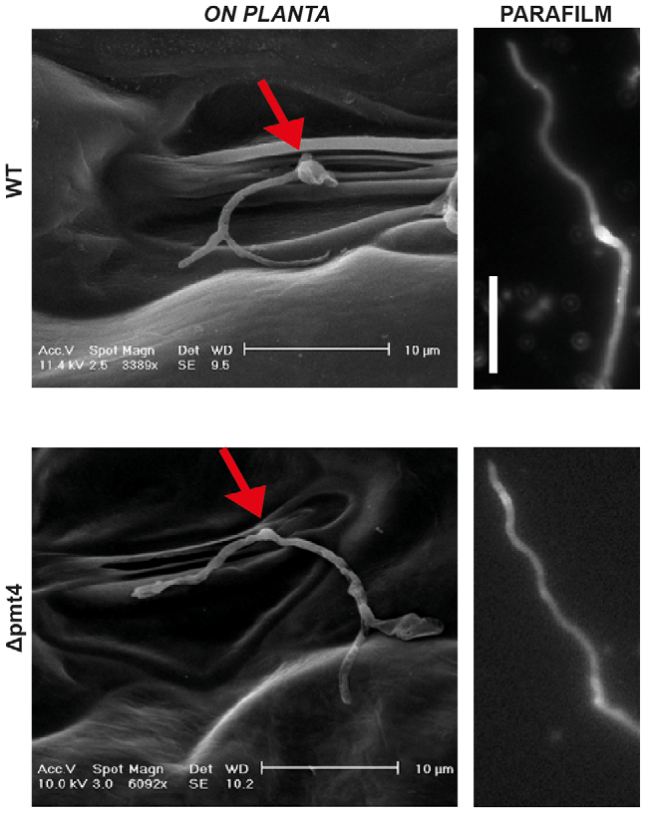
The *pmt4* mutant appressorium shows outgrowing as a consequence of its incapability to penetrate the plant cuticle. Crosses of the sexually compatible strains FB1 and FB2, and FB1Δpmt4 and FB2Δpmt4 were inoculated into maize seedlings. Leaves were fixed and analysed by Scanning Electron Microscopy (SEM) one day post infection. In these images (left), we can observe appressorium formation over a plant stomata (pointed with a red arrow) penetrating the plant surface in the wild-type. However, in a similar scenario, with the appressorium developed over plant stomata, the *pmt4* mutant is unable to penetrate the plant cuticle, producing hyphal outgrowing. This morphologic structure seems to be a consequence of its incapability to penetrate this surface as we can deduce from wild-type phenotype of the *in vitro* appressorium formation on the non-penetrable surface of parafilm M (right). The *in vitro* analysis was performed by fluorescence microscopy staining the cells with calcofluor white 15 hours after being sprayed on parafilm M (see Methods for additional details). Scale bar on the *in vitro* conditions represents 20 µm.

We could previously observe that the penetration of Δpmt4 appressoria was arrested [11]. To find out more about this phenotype, we carried out an analysis of appressorium penetration of wild-type strains and *pmt4* mutants using scanning electron microscopy (SEM). With this aim, we infected maize plants with a mixture of the compatible FB1 and FB2 as well as compatible FB1Δpmt4 and FB2Δpmt4 strains. 15 hours after infection maize leaves were fixed and visualized by SEM. In infections with compatible wild-type strains filaments differentiated into appressoria and penetrated inside the plant tissue. By contrast, the few appressoria formed by the Δpmt4 strain were unable to penetrate the cuticle (Figure 7), and instead the hyphae continued to grow on the plant surface. In order to verify that this aberrant morphology is a consequence but not the cause of the deficiency to penetrate the plant cuticle, we studied appressorium formation of FB1 and FB2, as well as FB1Δpmt4 and FB2Δpmt4 strains on a non-penetrable surface such as parafilm M. In the *in vitro* conditions post-appressorial outgrowth of hyphae was observed in both, *pmt4* mutants and wild-type strains, resembling the phenotype of Δpmt4 strains on the plant surface (Figure 7). This suggests that the Δpmt4 appressoria are unable to penetrate the plant cuticle and as consequence continue to grow on the plant surface.

### Pmt4 is required for cell adhesion in *U. maydis*

**Figure 8:**
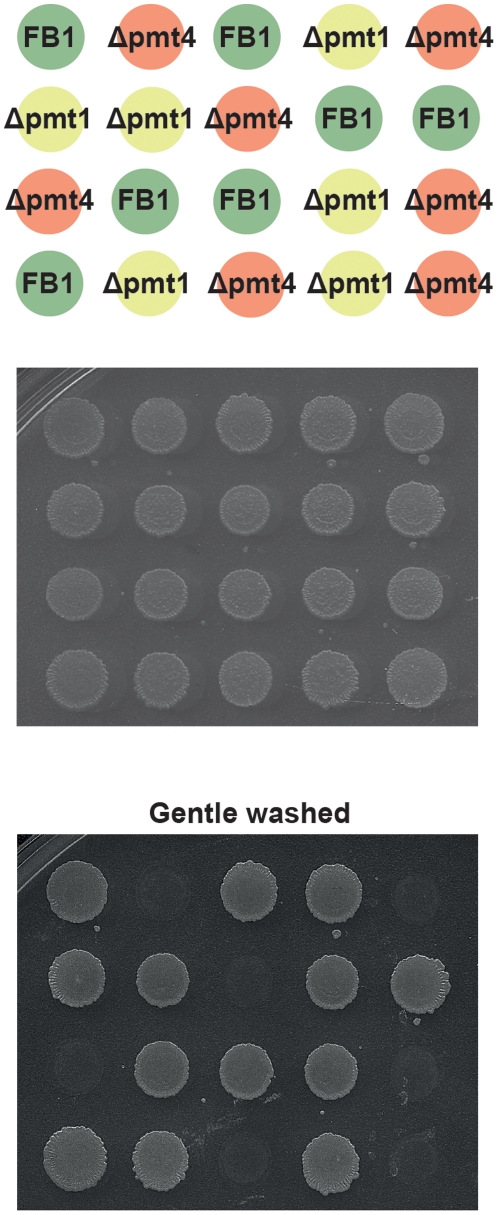
Pmt4 is required for cellular adhesion to solid surfaces in *U. maydis*. The strains indicated FB1 and FB1Δpmt4 were grown to A600 = 0.5 in YEPSL liquid medium and then were spotted on starch medium plates, according to the random distribution scheme shown, and incubated for three days at 28°C. Later, the plate surface was gentle washed. The FB1Δpmt1 strain was used as control of the effect of the loss of other protein O-mannosyltransferase. The deletion of *pmt4* reduces significantly the fungal cell adhesion to solid surfaces in *U. maydis*.

We have shown that the deletion of *pmt4* affects the perception of physical signals required for appressorium development as well as the appressorium-mediated penetration on plant surface. In the rice blast fungus *M. oryzae*, cell adhesion to surfaces has been shown to be needed for the detection of the physical plant-derived signals and for the penetration of the plant cuticle [49], [50]. Thus we wondered whether *pmt4* mutants were able to properly adhere to solid surfaces. To analyse adhesion capacity of Δpmt4 cells, we spotted exponentially growing cells of FB1 wild-type and FB1Δpmt4 strains on starch agar plates and incubated them for three days at 28°C. After this time period plates were washed with water. As shown in Figure 8, deletion of *pmt4* significantly reduces *U. maydis* cellular adhesion capacity since the colonies of FB1Δpmt4 were easily removed from the plates after a gentle washing. By contrast, wild-type colonies as well as Δpmt1 (control) colonies remained attached to the surface. This result shows that Pmt4 plays a role in cellular adhesion in *U. maydis* which may be connected to the appressorium formation and/or penetration defects. To distinguish between both possibilities, we studied the cellular adhesion capacity in FB1Δmsb2 strain. We observed a similar behaviour to wild-type cells (Figure S8). Because the loss of Msb2 affects to appressorium formation but not penetration, our data suggest a link between cellular adhesion capacity and appressorium penetration in *pmt4* mutant cells.

### Pmt4 plays a role after appressorium penetration

**Figure 9:**
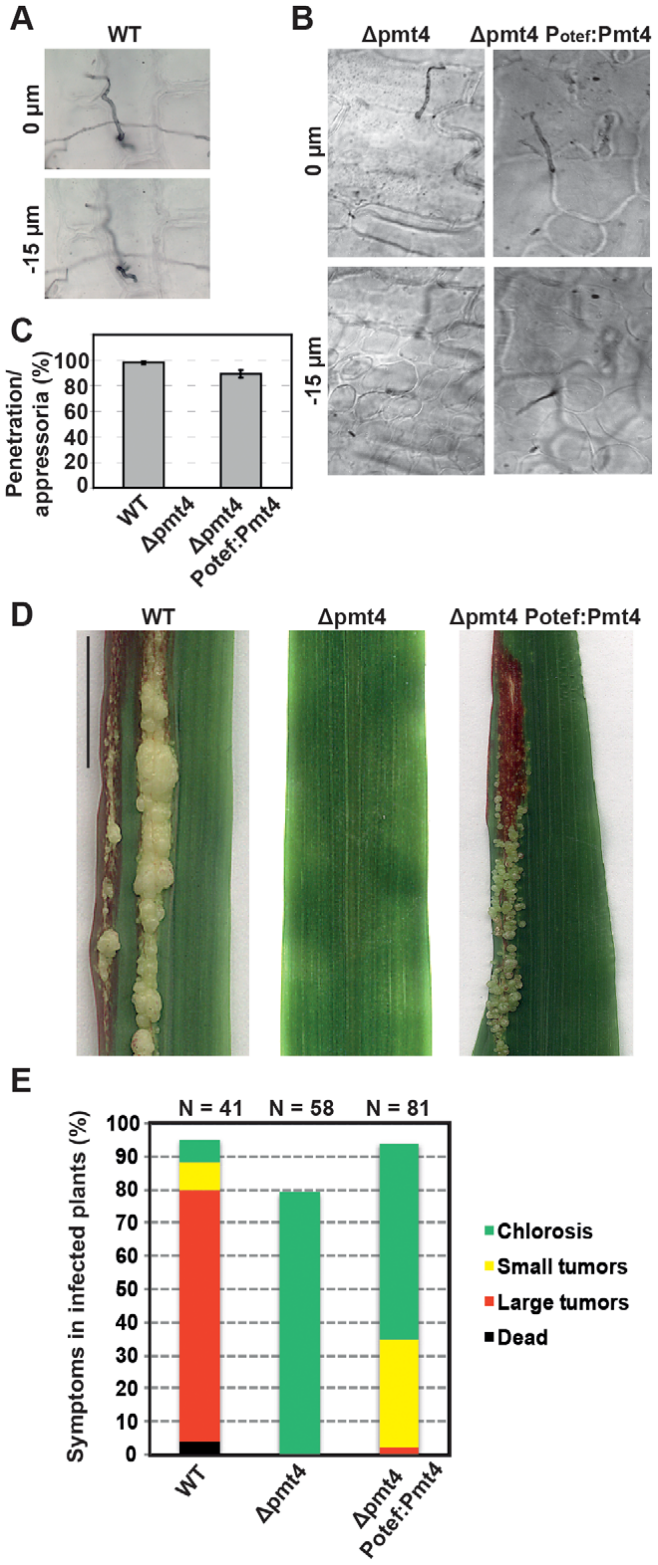
Pmt4 plays a role in the fungal proliferation inside the plant tissues. **A–B.** Seven days old maize seedlings were inoculated with SG200 (WT), SG200Δpmt4 and SG200Δpmt4P_otef_:pmt4. To check the appressorium penetration phenotype plant leaves were stained with chlorazol black two days after infection. The *z* axis image projections show the site of penetration (0 µm) and the hyphae invading into plant cells (−15 µm). **C.** Quantification of appressorium penetration. Data represent the mean values ±SEM. The number of appressoria analysed in each case was ≥30. **D.** 12 days after infection anthocyanin production and tumor formation were observed on plants infected with the wild-type while no infection symptoms were detected on plants inoculated with the *pmt4* mutant strain. The SG200Δpmt4P_otef_:pmt4 induces anthocyanin production and small tumors formation but not prominent tumors which suggest a role of Pmt4 after plant penetration. Bar represents 2 cm. **E.** Three independent experiments were performed 12 days post-infection and the average values are expressed as a percentage of the total number of infected plants (indicated by *N*). The strains analysed are shown below each column.

We have observed that although the plant infections with the SG200fuz7DDΔpmt4 strain were able to induce small tumors, the virulence of this strain was severely reduced when compared to SG200fuz7DD suggesting a role for Pmt4 during fungal progression inside the plant tissues. To confirm this result, we cloned the *pmt4* ORF under the control of the *otef* promoter which is active during early pathogenic development, but turned off during the late biotrophic stage [51]. This construct was integrated in the *ip locus* of the SG200Δpmt4 strain. To address if Pmt4 plays a role after plant penetration, we inoculated maize plants with SG200, SG200Δpmt4 and SG200Δpmt4P_otef_:pmt4. Disease progression was scored 12 days after infection. Although appressorium penetration was completely restored in plants infected with SG200Δpmt4P_otef_:pmt4, tumor formation was severely reduced compared to infections with SG200 (Figure 9). This demonstrates, that Pmt4 is not only needed for early pathogenic development, but has additional roles during the *in planta* development, most likely by posttranslational modification of fungal effectors required for tumor formation.

## Discussion

In this paper we have performed *in silico* screening for putative Pmt4 target proteins in the phytopathogen *U. maydis*. We have found a high percentage of proteins containing Ser/Thr rich regions with most of them having unknown functions. A screening approach searching for transmembrane anchored proteins, containing a stretch of 20 aa in which the percentage of Ser/Thr was ≥40%, identified 826 proteins (12.17% of the *U. maydis* proteome). By contrast, using similar parameters in *S. cerevisiae* 51 candidates (0.87% of the proteome) could be identified [8]. This difference may be due to the high number of putative secreted proteins present in the *U. maydis* proteome, which may be linked to the biotrophic relationship with the host plant [25]. In order to obtain a more restrictive group of Pmt4 target proteins we screened the proteome of *U. maydis* for single-pass transmembrane proteins containing a Ser/Thr-rich region facing the ER lumen. Under these conditions, we found 64 proteins. The validation of our screening retrieved novel Pmt4 substrates such as Um04580, or Pit1, which is essential for pathogenic development of *U. maydis* [38]. Moreover, we have found that Um03749, which belongs to the cluster 10A, required for tumor formation [25], is also a novel Pmt4 target protein.

One of the most intriguing phenotypes associated with the *pmt4* deletion in *U. maydis* is the strong reduction in appressorium formation and appressorium-mediated penetration of the plant cuticle [11]. Both processes are controlled by a MAP kinase pathway, consisting of the MAPKK kinase Kpp4/Ubc4, the MAPK kinase Fuz7/Ubc5 and the MAP kinases Kpp2/Ubc3 and Kpp6 [28]–[30], [32]–[34]. While Kpp2 is needed for appressorium formation [30], Kpp6 has a specific role during the penetration process [29]. Our results suggest that the role of Pmt4 during appressorium formation consists probably not in the glycosylation of downstream components of this pathway rather Pmt4 modifies proteins placed upstream of the MAP kinase pathway. In *U. maydis* the signalling mucin Msb2 is likely an activator of the MAP kinase cascade and the deletion of *msb2* leads to a strong reduction in appressorium formation due to defects in perception of plant surface signals [31]. We have identified Msb2 in our *in silico* screening and confirmed that it is substrate of Pmt4 in *U. maydis*, as it has been described for *S. cerevisiae* [44]. Our epistatic studies comprising *msb2* and *pmt4* suggest that during appressorium development both proteins act in the same pathway. A critical question was if Pmt4 and Msb2 are needed for the activation of the MAP kinase Kpp2, which is essential for filamentation and appressorium formation in *U. maydis* [30]. Here we could show for the first time that Kpp2 is phosphorylated during appressorium formation in *U. maydis*. However, in *pmt4* and *msb2* mutants phosphorylation of Kpp2 was only slightly reduced. In view of the fact that under our experimental conditions only a minor fraction of *U. maydis* filaments differentiate appressoria and that *pmt4* and *msb2* mutants exhibit normal filament formation, a process that depends also on Kpp2, we did not expect major differences in Kpp2 phosphorylation. We do currently not know in which spatial and temporal context Kpp2 phosphorylation induces either filament formation or appressorium formation. It is conceivable that appressorium formation requires a temporal coordinated threshold of MAP kinase activity and that this process involves Msb2 and its glycosylation status. A strong indication that Msb2 and Pmt4 act above the MAP kinase cascade is inferred from our observation that expression of the constitutive active *fuz7DD* allele restored appressorium formation in the Δmsb2 as well as Δpmt4 strains. Overall our data suggest that Msb2 and Pmt4 are needed for the activation of the MAP kinase pathway, although formal proof for this needs further experimentation.

In gel mobility shift analysis we found that Pmt4 specifically glycosylates the extracellular domain of Msb2. In *S. cerevisae* the extracellular domain of Msb2p has a negative regulatory function, since deletion of this domain leads to the activation of the FG (filamentous growth)-pathway specific MAP kinase Kss1p [46]. It is therefore assumed, that in yeast the release of the extracellular glycosylated domain of Msb2p activates the FG-pathway [45], [46]. This release can be mimicked by the deletion of *pmt4*, which leads to underglycosylated Msb2p and to the activation of the FG-pathway in an Msb2p-dependent manner [44]. Thus, in *S. cerevisiae* underglycosylated Msb2p activates the MAP kinase pathway. Our data from *U. maydis* demonstrates that the extracellular domain of Msb2 has a positive function, since deletion of the Ser/Thr-rich region causes a decrease in pathogenicity similar to the deletion of the entire *msb2* gene. This observation is consistent with the reduced appressorium formation in *pmt4* deletion mutants. Accordingly, Pmt4-mediated mannosylation of Msb2 seems to be necessary for activation of the MAP kinase cascade and the reduced virulence of Δpmt4 strains can be partially explained by defective glycosylation of the Msb2 sensor. Recent studies showed that the Ser/Thr-rich extracellular domain of *M. oryzae* Msb2 is like in *U. maydis* Msb2 and in contrast to Msb2p from *S. cerevisae* also essential for function [42]. This suggests that the model proposed for the Pmt4-Msb2 relationship, regulating appressorium development is conserved among phytopathogenic fungi (see Figure S9).

**Figure 10:**
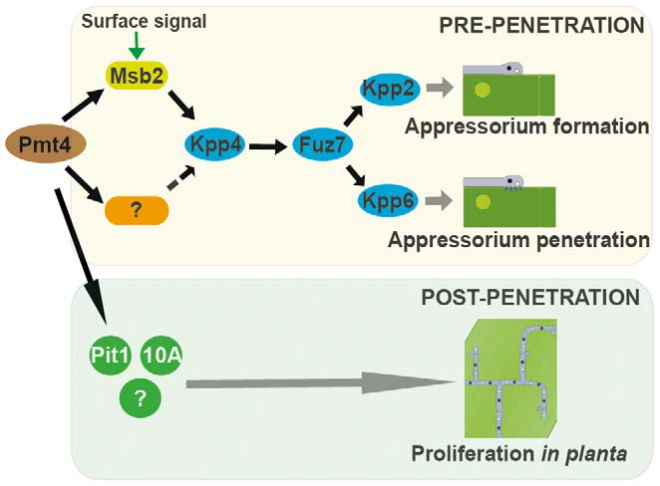
Model of the Pmt4 roles developed during *U. maydis* pathogenic development. Pmt4 has a major function in appressorium biology: Pmt4 O-mannosylates Msb2, a membrane-sensor that regulates appressorium development upstream of the MAP kinase cascade. The deletion of *pmt4* severely affects appressorium formation, probably due to a deficient glycosylation of Msb2. During appressorium-mediated penetration Pmt4 also probably acts above the MAP kinase cascade. Furthermore, Pmt4 plays a role *in planta* by O-mannosylation of proteins required for tumor formation.

The defect in appressorium formation can be explained by a defective Msb2 glycosylation. However, since the few appressoria produced in Δmsb2 strains are not affected in penetration [31], the penetration defect of Δpmt4 appressoria is likely caused by insufficient glycosylation of yet unidentified Pmt4 target proteins. Our observations using scanning electron microscopy showed that the few appressoria produced by the Δpmt4 strain were unable to penetrate the plant cuticle and instead continued growth of the hyphae on the plant surface. This phenotype has also been observed in strains carrying a non-phosphorylatable allele of the MAP kinase Kpp6 [29]. These findings, together with the observation that expression of the *fuz7DD* allele restores the penetration defect of Δpmt4 appressoria suggest that Pmt4 target proteins implicated in appressorium-mediated penetration are also likely to be placed upstream of the MAP kinase pathway (Figure 10).

To better analyse the outgrowing of the hypha observed in the *pmt4* mutant, we studied appressoria developed by wild-type strains on non-penetrable surfaces such as parafilm M, finding a similar behaviour. This data strongly suggest that this hyphal outgrowth is the consequence but not the cause of its incapability to penetrate the plant cuticle. The penetration failure of Δpmt4 appressoria could be caused by an insufficient adhesion of the fungus to the plant surface. For the rice blast fungus *M. oryzae* it has been demonstrated that proper adhesion is critical for appressoria to penetrate [50]. Interestingly, we have shown that Pmt4 plays an important role in cellular adhesion to surfaces which might be related to the appressorium penetration defect.

Our previous studies as well as this work demonstrated a prominent role of Pmt4 in the appressorium biology. In addition, we have now shown that Pmt4 is also necessary for tumor induction *in planta*. We observed that *pmt4* expression under the control of the *otef* promoter, which is turned off during the infection process [51], is not sufficient to restore normal tumor formation in the SG200Δpmt4 strain. Likewise, expression of *fuz7DD* in Δpmt4 strains did not rescue the virulence defect, although initial appressorium formation and penetration were restored. This suggests that the Pmt4 protein is also required during fungal proliferation inside the plant tissue. For *C. albicans* it has been demonstrated that the deletion of *pmt* genes is associated with defects in cell wall integrity and secretion of fungal effectors, probably disturbing the interaction with the host [5], [10], [52], [53]. In *U. maydis*, the biotrophic interaction between the plant and the fungus is also mediated by fungal effectors [24]. The role of Pmt4 during this developmental stage could be related to the O-mannosylation of Pit1 and Um03749 (see Figure 10), since both are important for the biotrophic development [25], [38]. Um03749 is a putative secreted protein which is directly modified by Pmt4. The underglycosylation of Um03749 in Δpmt4 strains might affect the function of this effector. Pit1 is a plasma membrane protein that is genetically linked to the secreted effector Pit2. Both proteins are specifically needed for tumor formation [38]. Although the connection between these two proteins is currently unclear, it is assumed, that they act in the same pathway [38]. Therefore, mannosylation of Pit1 by Pmt4 could indirectly affect the function of the secreted effector Pit2. This points to a role of Pmt4 in the regulation of secreted effector proteins, needed for the biotrophic development. In this context, considering the large number of putative Pmt4 targets obtained in this study, it is tempting to speculate that more Pmt4 targets exist, which contribute to the establishment of biotrophic interaction between plant and fungus.

The bioinformatic search for Pmt4 target proteins in other pathogenic fungi might highlight the role of Pmt4 during fungal pathogenic development and allow the identification of novel Pmt4 substrates required for fungus-host interactions.

## Materials and Methods

### Strains, growth conditions and plasmids

*Escherichia coli* DH5α and pGEM-T easy (Promega) were used for cloning purposes. pING, a derivative of pONG [31] where *mcherry* is exchanged by *gfp*, was used for cloning of ORFs from *pmt4*, *um03746*, *um03749* and *um04580*. To clone these ORFs, *U. maydis* genomic DNA was used as template amplifying with the primers Pmt4ORF-5 and Pmt4ORF-3, 03746ORF-5 and 03746ORF-3, 03749ORF-5 and 03749ORF-3, 04580ORF-5 and 04580ORF-3, respectively (sequences in Table S4). The fragments were cut with *Sfi*I and ligated with the 6.3 kb *Sfi*I fragment of pING. Expression of ORFs from pING derivatives is controlled by the *otef* promoter.

To generate the *msb2-HA-GFP* construct, the primer combinations oDL81/oDL125 and oDL82/oDL124 (Table S4) using *U. maydis* genomic DNA as template generated two PCR products, 1.4 kb and 2.1 kb in length, respectively. Both fragments were cut with *Sfi*I and ligated with the 6.3 kb *Sfi*I fragment of pING, resulting in pPotef-msb2-HA-GFP. In this plasmid the *msb2* gene with an internal HA tag (corresponding to amino acid 709) is C-terminally fused to GFP. Expression of the fusion gene is driven by the *otef* promoter.

To generate the truncated msb2ΔSTR-GFP allele, the primer combinations oDL124/oDL171 and oDL204/oDL125 using pPotef-msb2-HA-GFP as template generated two PCR products, 0.1 kb and 1.3 kb in length, respectively. Both fragments were cut with *Sfi*I and *Xma*I and ligated with the 6.3 kb *Sfi*I fragment of pING resulting in pPotef-msb2ΔSTR-GFP. In this plasmid the entire Ser/Thr-rich region of *msb2*, corresponding to amino acids 33 to 713 is deleted. All plasmids were linearized with *Ssp*I and integrated into the *ip locus*.

*U. maydis* strains used in this study are listed in Table 1. Deletion constructs were generated as described previously [25]. To generate single deletion mutants of *um04580* and *pmt4* genes, fragments of the 5′ and 3′ flank of their open reading frames were generated by PCR on *U. maydis* FB1 genomic DNA with the following primer combinations: Um04580KO5-1/Um04580KO5-2 and Um04580KO3-1/Um4580KO3-2; UmPMT4KO5-1/UmPMT4KO5-2 and UmPMT4KO3-1/UmPMT4KO3-2; (Sequences in Table S4). These fragments were digested with *Sfi*I and ligated with the 1.4 kb *Sfi*I nourseothricin (*ClonNAT*) resistance cassette [54].

**Table 1:** *U. maydis* strains used in this study.

Strain	Relevant Genotype	Reference
FB1	a1 b1	Banuett and Herskowitz (1989)
FB2	a2 b2	Banuett and Herskowitz (1989)
SG200	a1 mfa2 bW2 bE1	Bölker *et al.* (1995)
UMG1	a1 b1 Δpmt1	Fernández-Álvarez *et al.* (2009)
UMG4	a1 b1 Δpmt4	Fernández-Álvarez *et al.* (2009)
UMG5	a2 b2 Δpmt4	Fernández-Álvarez *et al.* (2009)
UMG19	a1 mfa2 bW2 bE1 Δpmt4	Fernández-Álvarez *et al.* (2009)
FB1Δmsb2	a1 b1 Δmsb2	Lanver *et al.* (2010)
SG200Δmsb2	a1 mfa2 bW2 bE1 Δmsb2	Lanver *et al.* (2010)
SG200AM1	a1 mfa2 bW2 bE1 P*_um01779_*:3xGFP	Mendoza-Mendoza *et al.* (2008)
SG200AM1Δmsb2	a1 mfa2 bW2 bE1 P*_um01779_*:3xGFP Δmsb2	Lanver *et al.* (2010)
SG200Δpit1/pit1-GFP	a1 mfa2 bW2 bE1Δpit1 P*_otef_*:pit1-GFP	Doehlemann *et al.*, (2011)
UMG25	a1 mfa2 bW2 bE1 P*_otef_*:um03746-GFP	This work
UMG26	a1 mfa2 bW2 bE1 P*_otef_*:um03746-GFP Δpmt4	This work
UMG27	a1 mfa2 bW2 bE1 P*_otef_*:um03749-GFP	This work
UMG28	a1 mfa2 bW2 bE1 P*_otef_*:um03749-GFP Δpmt4	This work
UMG29	a1 mfa2 bW2 bE1 P*_otef_*:um04580-GFP	This work
UMG30	a1 mfa2 bW2 bE1 P*_otef_*:um04580-GFP Δpmt4	This work
UMG31	a1 mfa2 bW2 bE1 Δum04580	This work
UMG33	a1 mfa2 bW2 bE1 Δpit1 P*_otef_*:pit1-GFP Δpmt4	This work
SG200Δmsb2/msb2-HA-GFP	a1 mfa2 bW2 bE1 Δmsb2 P*_otef_*:msb2-HA(aa709)-GFP	This work
UMG35	a1 mfa2 bW2 bE1 Δmsb2 P*_otef_*:msb2-HA(aa709)-GFP Δpmt4	This work
UMG36	a1 mfa2 bW2 bE1 P*_um01779_*:3xGFP Δpmt4	This work
UMG37	a1 mfa2 bW2 bE1 P*_um01779_*:3xGFP Δmsb2Δpmt4	This work
UMG38	a1 mfa2 bW2 bE1 P*_crg_*:fuz7DD	This work
UMG39	a1 mfa2 bW2 bE1 P*_crg_*:fuz7DD Δpmt4	This work
UMG67	a1 mfa2 bW2 bE1 Δpmt4 P*_otef_*:pmt4	This work
UMG68	a1 mfa2 bW2 bE1 Δmsb2Δpmt4	This work
UMG69	a1 mfa2 bW2 bE1 Δpit1 P*_otef_*:pit1-GFP Δcwh41	This work
UMG70	a1 mfa2 bW2 bE1 Δmsb2 P*_otef_*:msb2-HA(aa709)-GFP Δcwh41	This work
UMG71	a1 mfa2 bW2 bE1 P*_crg_*:fuz7DD Δmsb2	This work
SG200Δmsb2/msb2ΔSTR-GFP	a1 mfa2 bW2 bE1Δmsb2 P*_otef_*:msb2(Δ33-713)-GFP	This work

Wild-type *U. maydis* strains FB1 (*a1 b1*) and FB2 (*a2 b2*) [55], solopathogenic strain SG200 (*a1:mfa2 bE1 bW2*) [56] cells were grown at 28°C in liquid YEPSL (0.4% bactopeptone, 1% yeast extract and 0.4% saccharose) medium.

Pathogenicity assays were performed as described [25]. *U. maydis* cultures were grown to exponential phase and concentrated to an OD_600_ of 3, washed two times in water and injected into one week old maize (*Zea mays*) seedlings (Golden Bantam). Disease symptoms were quantified 7 to 25 days post infection. Virulence tests were repeated at least three times using the number of plants indicated in each figure.

### DNA procedures

Molecular biology techniques were used as described by [57]. *U. maydis* DNA was isolated following the protocol of [58]. Standard *U. maydis* transformation procedure was used [59].

### *In silico* screening analysis

The *U. maydis* proteome was downloaded from the MIPS FTP server at ftp://ftpmips.gsf.de/ustilago/Umaydis_valid/Umaydis_valid_orf_prot_121009, and the identifiers of proteins annotated as transmembrane were downloaded from the MIPS web server. A program to filter the protein sequences was written using the Perl programming language. The program searches for proteins with a fixed frequency of Ser/Thr within a window of selected length. The most restrictive screening was carried out selecting only transmembrane proteins, avoiding GPI-anchored and multipass membrane proteins.

### Western blot analysis

Protein extracts were prepared from exponentially growing cells, collected by centrifugation, washed with stop buffer (0.9% NaCl, 1 mM NaN3, 10 mM EDTA, 50 mM NaF), and frozen on dry ice. All manipulations were done on ice or in the cold room (4°). For isolation of total protein extracts from surface-attached hyphae, cell suspensions were incubated on parafilm M as described below. Cells not attached to the surface were removed in a water bath. Attached hyphae were harvested in Thorner buffer (8 M urea, 5% SDS, 0.1 mM EDTA, 0.01% bromophenol blue, 100 mM DTT, and 100 mM Tris-HCl, pH 6.8) using a cell scraper (Greiner, Frickenhausen/Germany). Total protein extracts were prepared by Fast-prep vortexing with glass beads (Sigma) [60]. For isolation of secreted proteins, *U. maydis* cells were grown in 50 mL of CM medium supplemented with 1% glucose to an OD 600 of 0.4. Free-cell culture supernatant was obtained by centrifugation at 3.000 g for ten minutes at 4°C. Secreted proteins were isolated by incubation for 30 minutes on ice with 0.02% sodium deoxycholate and further addition of 10% Trichloroacetic acid. Samples were incubated overnight at 4°C and precipitated proteins were collected by centrifugation at 12.000 g for 15 minutes at 4°C. The pellet was air dried and resuspended in 200 µl of loading buffer 2X (0.1 mM Tris-HCl pH 6.8, 4% SDS, 20% glycerol, 2.98 mM bromophenol blue, 0.2 M DTT). Purified proteins were separated on SDS–PAGE (6–12% of polyacrylamide). Blots were probed with anti-HA antibodies (Roche) or anti-GFP mouse IgG1 k antibodies (Roche). To detect phosphorylated Kpp2 anti-phospho-p42/44 antibody (cell signaling, Danvers/USA) was used. To detect total Kpp2 a polyclonal anti-Kpp2 antibody was generated in rabbits using the Kpp2 antigen sequence N-CLTFSPRKRITVEEAL-C (Eurogentec, Cologne/Germany). Anti-alpha tubulin was routinely used as loading control. As secondary antibodies horseradish peroxidase–conjugated anti-mouse IgG (Sigma) or anti-rabbit IgG (Cell Signaling) were used. Supersignal (Pierce) was used to detect the proteins analysed. Quantification of western blots was performed using a chemocam imager (INTAS, Göttingen/Germany) and ImageJ software.

### Filament and appressorium induction on artificial surfaces

The *in vitro* system for inducing filaments and appressoria in *U. maydis* was applied as describes previously [27] with minor modifications [31]. Cell cultures (2% YEPSL) were sprayed (EcoSpray Labo Chimie, France) on Parafilm M (Pechiney Plastic Packaging, Chicago, USA) and, if applicable, treated with 100 µM (f.c.) 16-hydroxyhexadecanoic acid (Sigma). To quantify filaments relative to yeast-like cells samples were directly analysed by light microscopy. To quantify appressoria, surfaces were rinsed with water and later, stained with calcofluor white (1 µg ml^−1^) (Sigma). To quantify the production of appressoria, filaments stained with calcofluor white were counted relative to filaments showing eGFP fluorescence. Data are expressed as means ±SEM of triplicate samples. Statistical significance was assessed using Statistical Calculators (http://www.graphpad.com/quickcalcs/index.cfm) and considered significant if P values were <0.05.

### Scanning Electron Microscopy

The SEM analyses were performed on PHILIPS XL30 microscope. Infected leaves from maize two days post-infection were fixed with 4% glutaraldehyde in cacodylate (0.1 M pH 7.4) at 4°C. The samples were washed several times in cacodylate. Afterwards, a second fixation in 1% tetraoxide osmium was carried out for two hours at 4°C. This was washed in cacodylate containing 7.5% of sucrose. Dehydration with acetone at 4°C: 30 minutes with 70% acetone, 30 minutes with 80% acetone, 30 minutes 90% acetone and finally, one hour with 100% acetone. The samples were then introduced into an SPI-Dry Critical Point Drying apparatus and coated in gold.

### Cellular adhesion assays

To ascertain the effects of the deletion of *pmt4* and *msb2* on cellular adhesion, we grew the strains to exponential phase in YEPSL at 28°C and the cells were spotted on starch agar plates. Starch medium contains, 0.25% starch from potato, 0.1% ammonium sulphate, 0.1% sucrose and phosphate buffer 25 mM pH 7. Plates were incubated for three days at 28°C and cellular colonies were rinsed with water to analyse their adhesion properties.

### Calcofluor white and chlorazol black E staining

To analyze the pre-penetration stages of *U. maydis* using fluorescence microscopy, cells were stained with calcofluor white. Post-penetration stages were studied by optical microscopy of chlorazol black E stained leaf samples as previously described [29].

### Microscopy

Cells were examined using a Leica fluorescence microscope, equipped with a PlanApo ×100 lens. Analysis of the pre-penetration stages was done using a Deltavision widefield microscope (Applied Precision, Issaquah, WA). Image deconvolution was performed using z-series of between 7 and 23 focal planes, acquired at 0.5 µm intervals. Image processing was carried out using Adobe Photoshop CS5 and ImageJ.

### Accession numbers

*U. maydis* sequence data can be found in the UniProt data library under accession numbers UniProt:Q4P380 for Pmt4, UniProt:Q4P140 for Pmt1, UniProt:Q4PAX9 for Cwh41, UniProt:Q4PHD3 for Msb2, UniProt:Q9UQY5 for Kpp2/Ubc3, UniProt:Q4PC32 for Kpp6, UniProt:Q8J230 for Kpp4/Ubc4, UniProt:Q99078 for Fuz7/Ubc5, UniProt:Q4PET9 for Pit1, UniProt:Q4PF78 for Um01235, UniProt:Q4P817 for Um03746, UniProt:Q4P814 for Um03749 and UniProt:Q4P5N3 for Um04580.

## Supporting Information

Figure S1 **The** ***U. maydis*** **life's pathogenic cycle.** The sexual pathogenic cycle of *U. maydis* starts with the mating between two sexually compatible strains on the plant surface to form a dikaryon filament. A set of combined physical-chemical plant-derived signals leads to hyphae differentiation into appressoria which mediate plant penetration (1). Once inside the plant, the fungus proliferates as mycelium developing the clamp-like cells which ensure the maintenance of the dikaryotic state (2). During this infection process, *U. maydis* induces the tumor formation in the plant maize (3). Pmt4 is required for appressorium formation and penetration, and thus the Δpmt4 strain is unable to proliferate inside the plant tissues neither induce tumors.
(TIFF)

Figure S2 **Enrichment analysis for FunCatDB (MUMDB MIPS) of Pmt4 putative target proteins.**
(TIFF)

Figure S3 **Um04580 is not required for** ***U. maydis*** **pathogenic development.** Plants were infected with the strains indicated and symptoms were scored 12 days post-infection. *N* indicates the total number of plants evaluated in each case.
(TIFF)

Figure S4 **Pit1 and Msb2 processing in the** ***cwh41*** **mutant.** **A.** Western blot analysis of Pit1 tagged with GFP in the SG200 (WT), SG200Δpmt4 and SG200Δcwh41 backgrounds. α-GFP antibody was used to detect the Pit1-GFP protein. We did not observe the bands that correspond to the glycosylated fraction of the protein in the *cwh41* mutant. Thus, Pit1 processing depends on protein N- and O-glycosylation pathways. **B.** Western Blot analysis of Msb2-HA-GFP isolated from SG200Δmsb2/msb2-HA-GFP (WT), SG200Δmsb2Δpmt4/msb2-HA-GFP and SG200Δmsb2Δcwh41/msb2-HA-GFP. α-HA antibody was used to detect the N-terminal part of Msb2. Equal amounts of proteins of total cell extracts were loaded in each lane. The deletion of *cwh41* does not affect significantly the Msb2 mobility.
(TIFF)

Figure S5 **N-terminal domain of** ***U. maydis*** **Msb2 is secreted.** Western Blot analysis of Msb2-HA-GFP isolated from SG200Δmsb2/msb2-HA-GFP culture supernatant (S) and total extract (TE). SG200 was used as a negative control. The first gel (above) was used to detect the N-terminal part of Msb2 with α-HA antibody. The other gel (below) was treated with α-GFP antibody to detect the C-terminus of Msb2. The extracellular N-terminal domain of *U. maydis* Msb2 was identified in the culture supernatant, while the C-terminal fragment was exclusively detected in the cellular fraction.
(TIFF)

Figure S6 **Expression of** ***fuz7DD*** **partially restores the** ***msb2*** **mutant phenotypes.** **A.** Seven days old maize seedlings were infected with the strains SG200 (WT), SG200fuz7DD, SG200Δmsb2 and SG200fuz7DDΔmsb2 scoring appressoria production 15 hours later (>100 filaments in each case). Data are shown as mean values ±SEM. Asterisk indicates statistically significant differences between wild-type (control) and Δmsb2 strains, P value≤0.001. **B.** Symptoms in infected plants with the strains indicated were scored 12 days post-infection. *N* indicates the total number of plants evaluated in each case.
(TIFF)

Figure S7 **Kpp2 phosphorylation in Δpmt4 and Δmsb2 strains.** **A.** The WT (SG200), Δpmt4 and Δmsb2 strains were incubated on parafilm M with 100 µM 16-hydroxyhexadecanoic acid for 10 h. Total proteins isolated before (left) and after incubation on parafilm M (right) were subjected to western blot analysis. The phosphorylated form of Kpp2 (P-Kpp2) and total Kpp2 were detected using α-phospho-p44/42 antibody and α-Kpp2 antibody, respectively. Asterisk denotes an unspecific background signal. **B.** The relative Kpp2 phosphorylation from three independent experiments. Kpp2 phosphorylation in WT (SG200) was set to 1. The datasets from Δpmt4 and Δmsb2 strains were compared with the wild-type dataset to calculate p-values (t-test) given above column. Error bars indicate standard deviation.
(TIFF)

Figure S8 **Msb2 is not required for cellular adhesion to solid surfaces.** The strains indicated were grown to A600 = 0.5 in YEPSL liquid medium and then were spotted on starch medium plates and incubated for three days at 28°C. The deletion of *msb2* does not affect the fungal cell adhesion to solid surfaces in *U. maydis*.
(TIFF)

Figure S9 **Working model of the possible divergent function of the Ser/Thr rich region of Msb2 in** ***S. cerevisiae*** **and phytopathogenic fungi such as** ***U. maydis*** **.** The conserved plasma membrane protein Msb2 acts upstream of MAP kinase cascades in fungi which regulates pseudohyphal growth in *S. cerevisiae* and appressorium development in *U. maydis*. In wild-type conditions (left) the extracellular domain of Msb2 is O-mannosylated by Pmt4. The absence of Pmt4 could have a divergent effect on the activation of the pathway in *S. cerevisiae* and *U. maydis* (see discussion).
(TIFF)

Table S1 **List of** ***U. maydis*** **proteins containing, at least, one plasma membrane anchoring and a window of 20 aa where the percentage of Ser/Thr is ≥40%.** The position of the window and the percentage on 1 is shown.
(XLS)

Table S2 **List of** ***U. maydis*** **proteins containing, at least, plasma membrane anchoring and a window of 40 aa where the percentage of Ser/Thr is ≥40%.** The position of the window and the percentage on 1 is shown.
(XLS)

Table S3 **List of** ***U. maydis*** **one trasmembrane proteins containing the window of 40 aa where the percentage of Ser/Thr is ≥40% in the luminal part.** The position of the window and the percentage on 1 is shown.
(XLS)

Table S4 **Primers used in this study.**
(DOC)
