# Supplementary figures and images for: Identification of O-mannosylated Virulence Factors in Ustilago maydis
Source: PLoS Pathog. 2012 Mar 1;8(3):e1002563. doi: 10.1371/journal.ppat.1002563 (PMC3295589; doi:10.1371/journal.ppat.1002563)

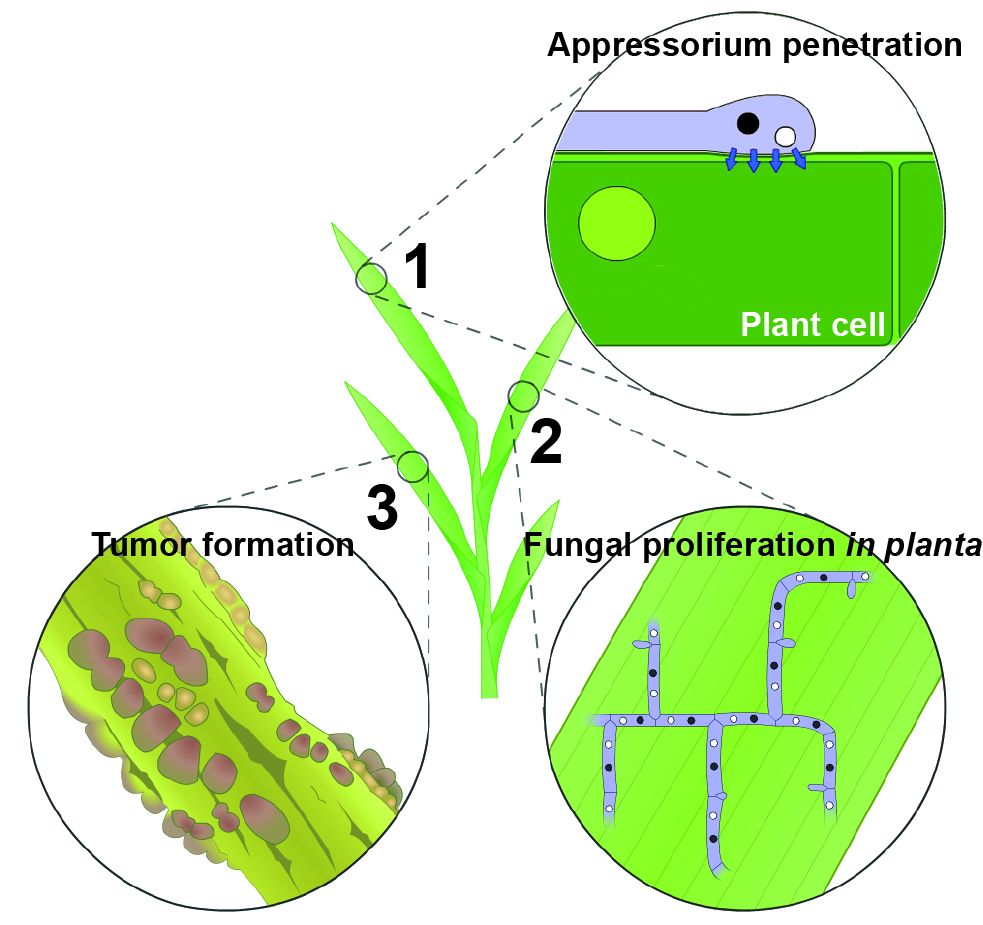

Supplement: Figure S1 — The U. maydis life's pathogenic cycle. The sexual pathogenic cycle of U. maydis starts with the mating between two sexually compatible strains on the plant surface to form a dikaryon filament. A set of combined physical-chemical plant-derived signals leads to hyphae differentiation into appressoria which mediate plant penetration (1). Once inside the plant, the fungus proliferates as mycelium developing the clamp-like cells which ensure the maintenance of the dikaryotic state (2). During this infection process, U. maydis induces the tumor formation in the plant maize (3). Pmt4 is required for appressorium formation and penetration, and thus the Δpmt4 strain is unable to proliferate inside the plant tissues neither induce tumors. (TIFF) [file ppat.1002563.s001.tiff]

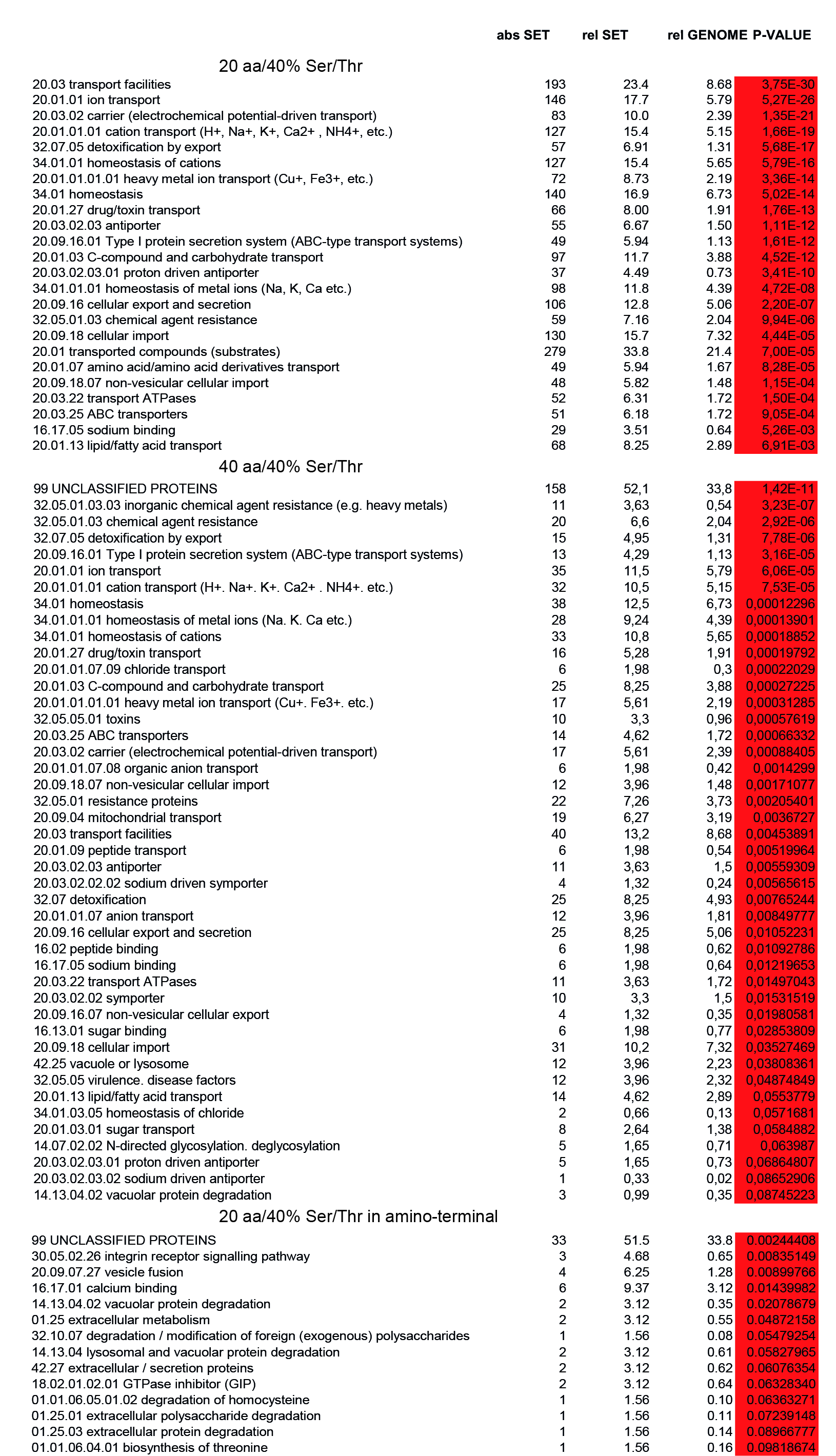

Supplement: Figure S2 — Enrichment analysis for FunCatDB (MUMDB MIPS) of Pmt4 putative target proteins. (TIFF) [file ppat.1002563.s002.tiff]

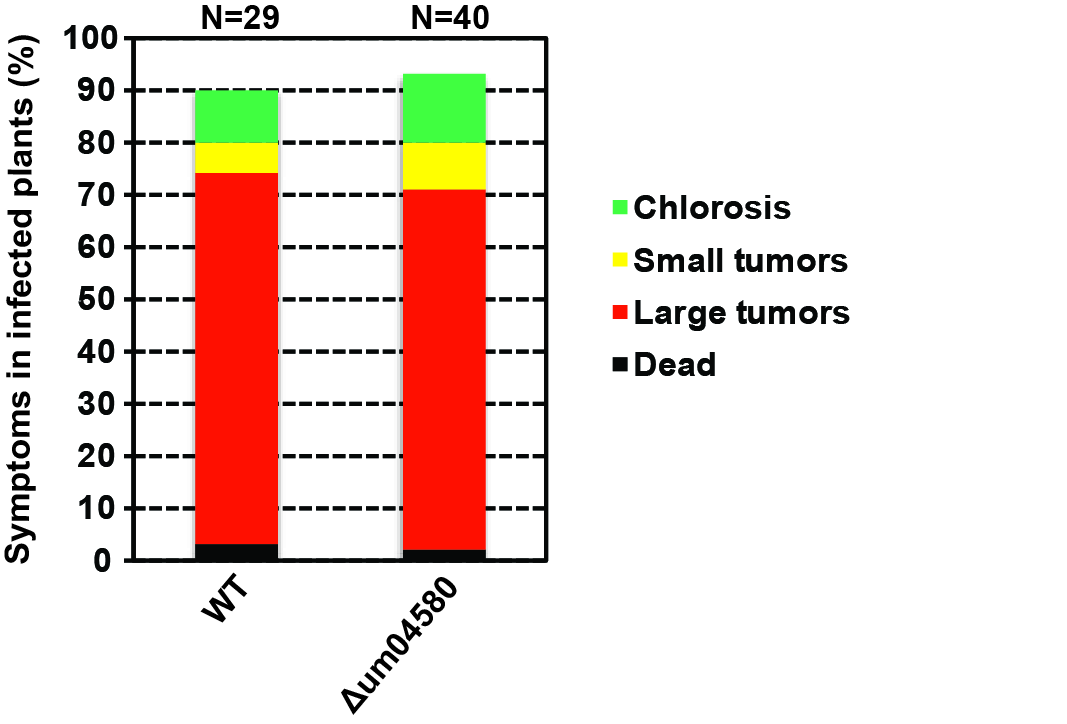

Supplement: Figure S3 — Um04580 is not required for U. maydis pathogenic development. Plants were infected with the strains indicated and symptoms were scored 12 days post-infection. N indicates the total number of plants evaluated in each case. (TIFF) [file ppat.1002563.s003.tiff]

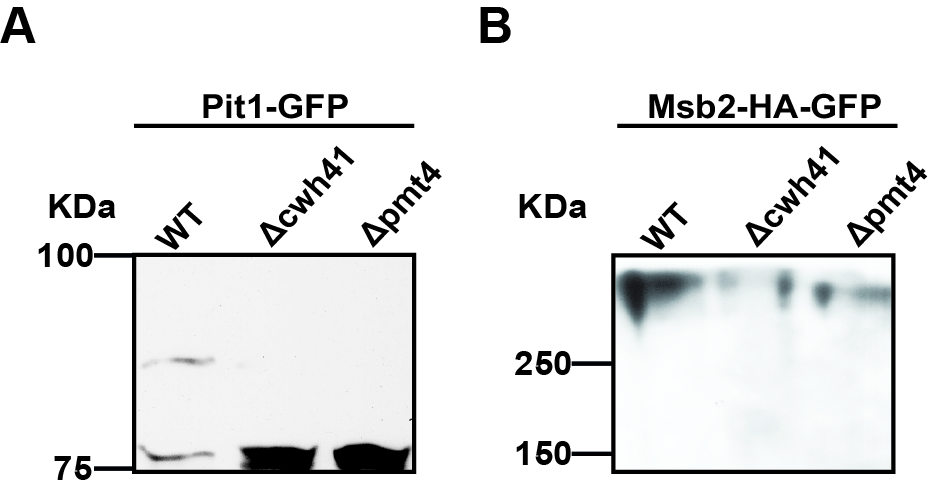

Supplement: Figure S4 — Pit1 and Msb2 processing in the cwh41 mutant. A. Western blot analysis of Pit1 tagged with GFP in the SG200 (WT), SG200Δpmt4 and SG200Δcwh41 backgrounds. α-GFP antibody was used to detect the Pit1-GFP protein. We did not observe the bands that correspond to the glycosylated fraction of the protein in the cwh41 mutant. Thus, Pit1 processing depends on protein N- and O-glycosylation pathways. B. Western Blot analysis of Msb2-HA-GFP isolated from SG200Δmsb2/msb2-HA-GFP (WT), SG200Δmsb2Δpmt4/msb2-HA-GFP and SG200Δmsb2Δcwh41/msb2-HA-GFP. α-HA antibody was used to detect the N-terminal part of Msb2. Equal amounts of proteins of total cell extracts were loaded in each lane. The deletion of cwh41 does not affect significantly the Msb2 mobility. (TIFF) [file ppat.1002563.s004.tiff]

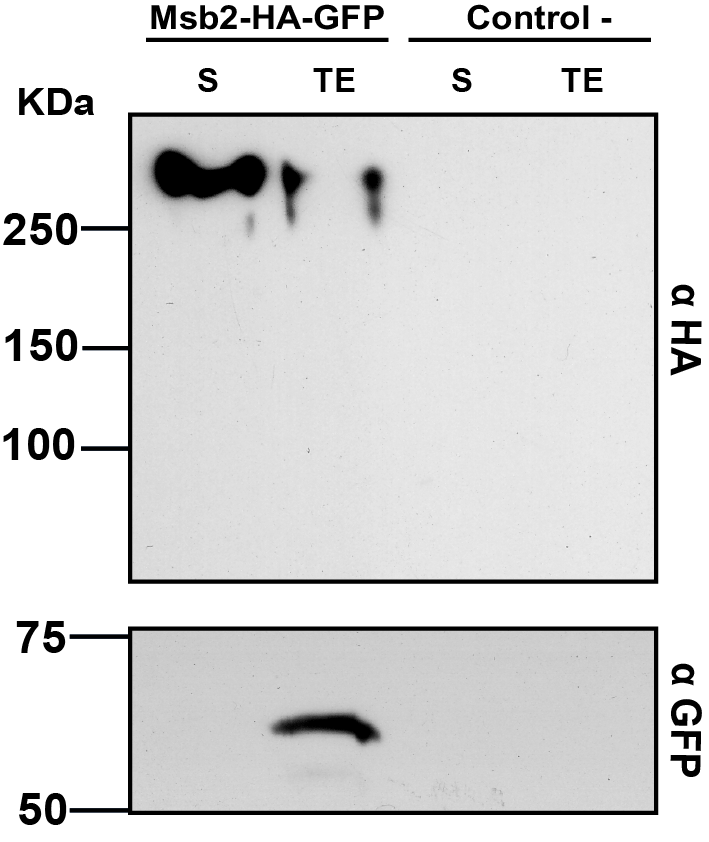

Supplement: Figure S5 — N-terminal domain of U. maydis Msb2 is secreted. Western Blot analysis of Msb2-HA-GFP isolated from SG200Δmsb2/msb2-HA-GFP culture supernatant (S) and total extract (TE). SG200 was used as a negative control. The first gel (above) was used to detect the N-terminal part of Msb2 with α-HA antibody. The other gel (below) was treated with α-GFP antibody to detect the C-terminus of Msb2. The extracellular N-terminal domain of U. maydis Msb2 was identified in the culture supernatant, while the C-terminal fragment was exclusively detected in the cellular fraction. (TIFF) [file ppat.1002563.s005.tiff]

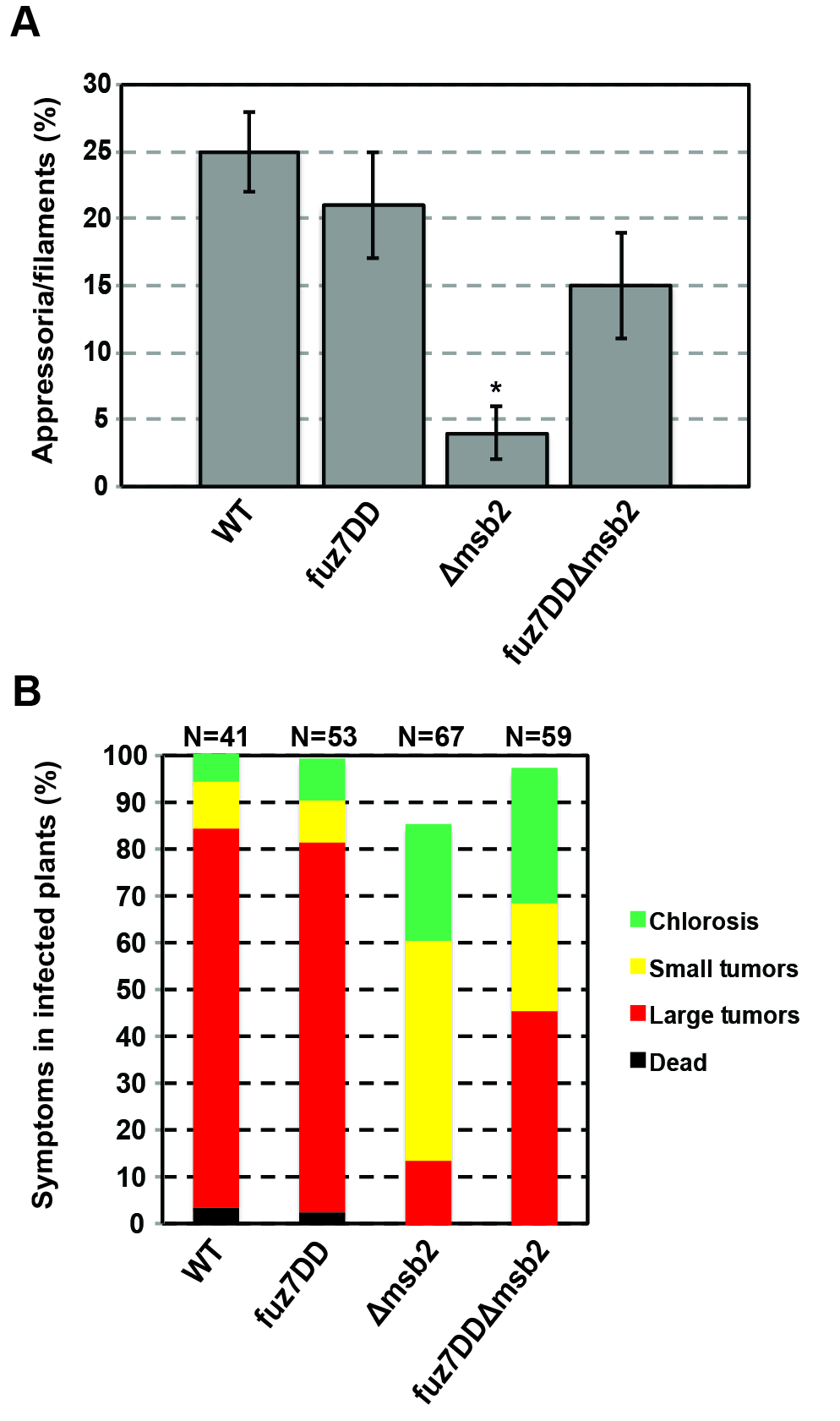

Supplement: Figure S6 — Expression of fuz7DD partially restores the msb2 mutant phenotypes. A. Seven days old maize seedlings were infected with the strains SG200 (WT), SG200fuz7DD, SG200Δmsb2 and SG200fuz7DDΔmsb2 scoring appressoria production 15 hours later (>100 filaments in each case). Data are shown as mean values ±SEM. Asterisk indicates statistically significant differences between wild-type (control) and Δmsb2 strains, P value≤0.001. B. Symptoms in infected plants with the strains indicated were scored 12 days post-infection. N indicates the total number of plants evaluated in each case. (TIFF) [file ppat.1002563.s006.tiff]

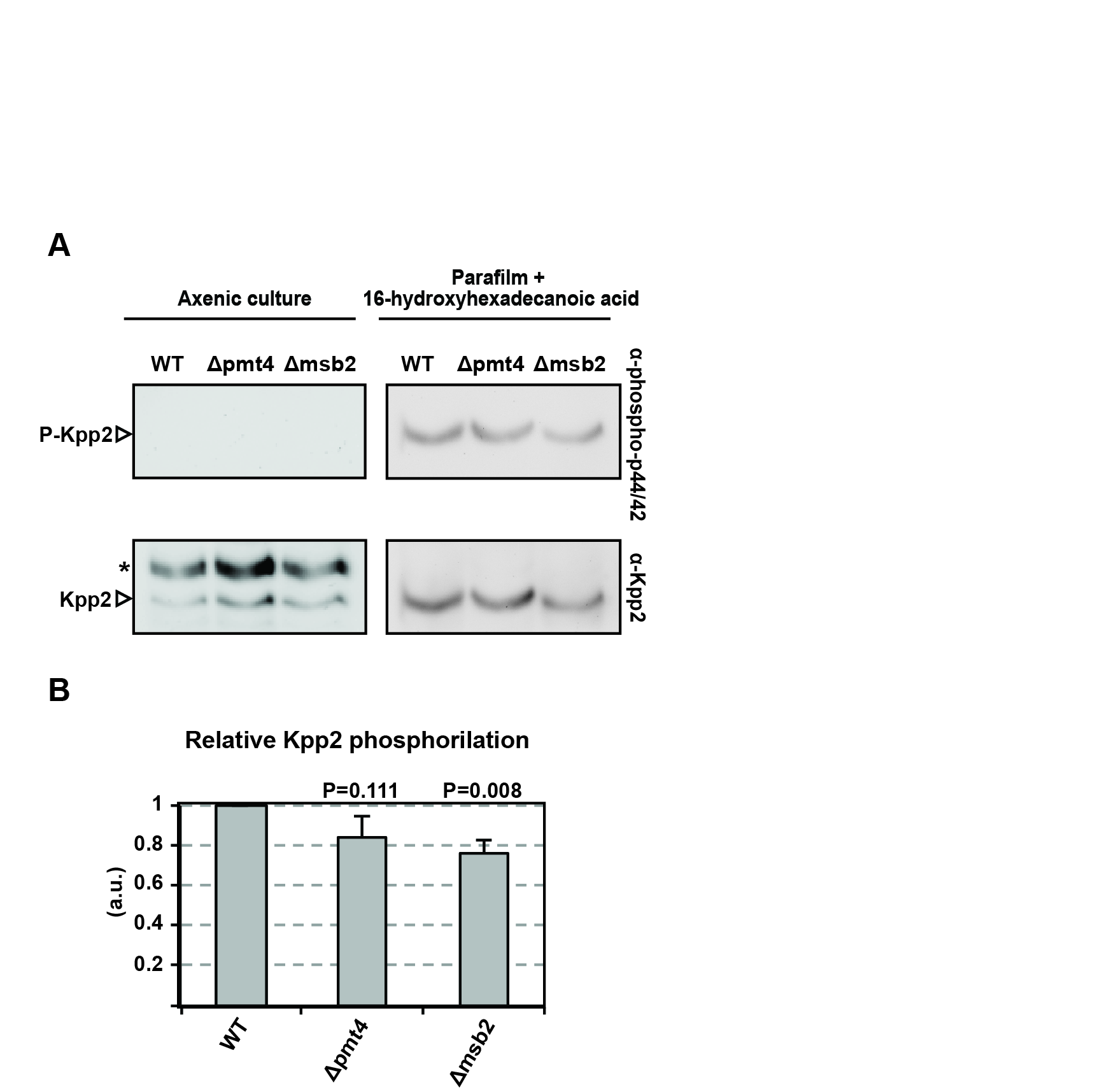

Supplement: Figure S7 — Kpp2 phosphorylation in Δpmt4 and Δmsb2 strains. A. The WT (SG200), Δpmt4 and Δmsb2 strains were incubated on parafilm M with 100 µM 16-hydroxyhexadecanoic acid for 10 h. Total proteins isolated before (left) and after incubation on parafilm M (right) were subjected to western blot analysis. The phosphorylated form of Kpp2 (P-Kpp2) and total Kpp2 were detected using α-phospho-p44/42 antibody and α-Kpp2 antibody, respectively. Asterisk denotes an unspecific background signal. B. The relative Kpp2 phosphorylation from three independent experiments. Kpp2 phosphorylation in WT (SG200) was set to 1. The datasets from Δpmt4 and Δmsb2 strains were compared with the wild-type dataset to calculate p-values (t-test) given above column. Error bars indicate standard deviation. (TIFF) [file ppat.1002563.s007.tiff]

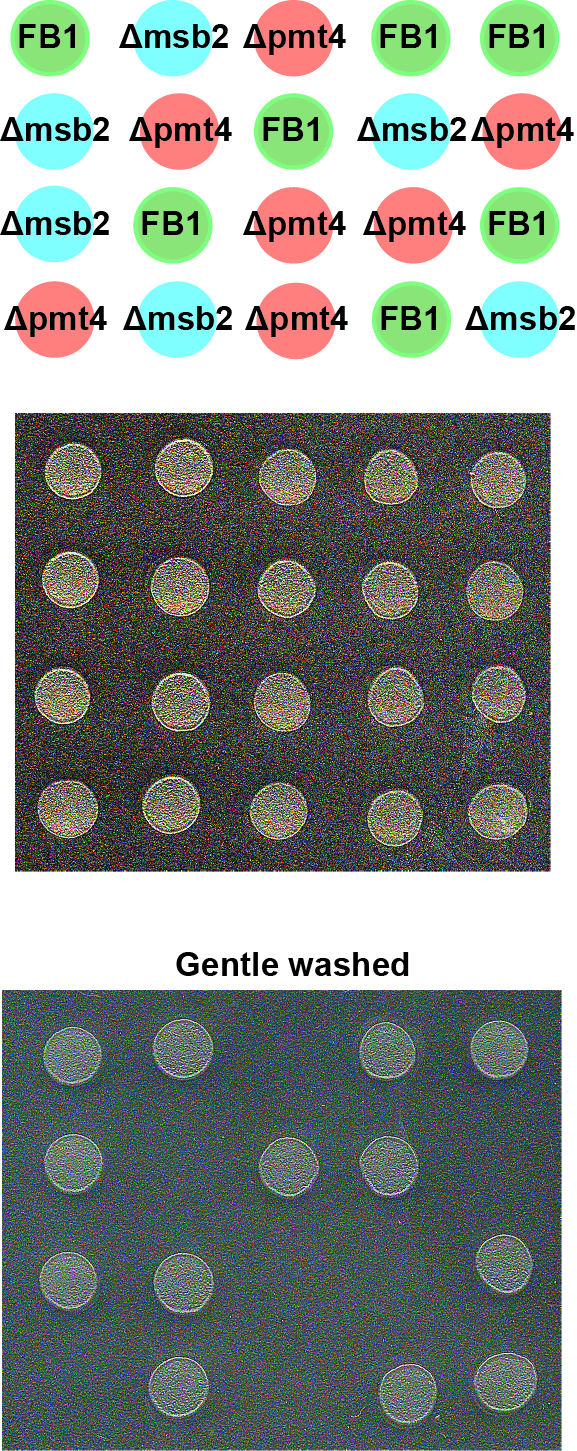

Supplement: Figure S8 — Msb2 is not required for cellular adhesion to solid surfaces. The strains indicated were grown to A600 = 0.5 in YEPSL liquid medium and then were spotted on starch medium plates and incubated for three days at 28°C. The deletion of msb2 does not affect the fungal cell adhesion to solid surfaces in U. maydis. (TIFF) [file ppat.1002563.s008.tiff]

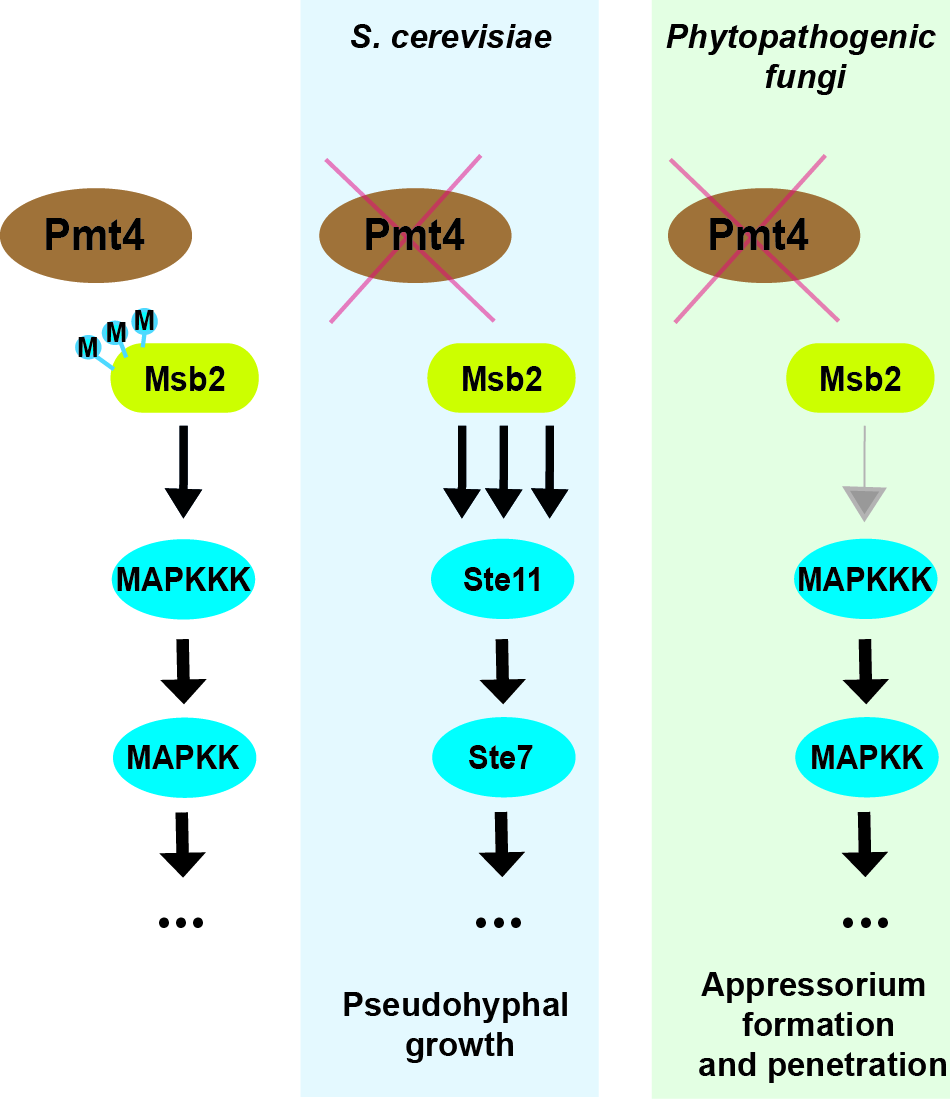

Supplement: Figure S9 — Working model of the possible divergent function of the Ser/Thr rich region of Msb2 in S. cerevisiae and phytopathogenic fungi such as U. maydis . The conserved plasma membrane protein Msb2 acts upstream of MAP kinase cascades in fungi which regulates pseudohyphal growth in S. cerevisiae and appressorium development in U. maydis. In wild-type conditions (left) the extracellular domain of Msb2 is O-mannosylated by Pmt4. The absence of Pmt4 could have a divergent effect on the activation of the pathway in S. cerevisiae and U. maydis (see discussion). (TIFF) [file ppat.1002563.s009.tiff]
