# Supplementary material for: Identification of O-mannosylated Virulence Factors in Ustilago maydis
Source: PLoS Pathog. 2012 Mar 1;8(3):e1002563. doi: 10.1371/journal.ppat.1002563 (PMC3295589; doi:10.1371/journal.ppat.1002563)
Supplement: Table S4 — Primers used in this study. (DOC) [file ppat.1002563.s013.doc]

| **Name** | **5’🡪 3’ Orientation** |
| --- | --- |
| PMT4KO5-1 | GCATCGGAAGTTGGTATGGC |
| PMT4KO5-2 | CACGGCCTGAGTGGCCCGTCAAACACTACCTGATCG |
| PMT4KO3-1 | GTGGGCCATCTAGGCATCCACCACTATCTCCCTGC |
| PMT4KO3-2 | ACAAGATCCTGCCACTCACC |
| 04580KO5-1 | TCGTGTTTGAGAATAATGCAAGGC |
| 04580KO5-2 | CACGGCCTGAGTGGCCAATGCGGTCCAGTCAAGGAAGGCC |
| 04580K03-1 | GTGGGCCATCTAGGCCACCGCCATCCTATTTGTTGAATGCC |
| 04580K03-2 | AGGCTCAGGTCTTACAATCCGTCG |
| 03746ORF-5 | ATAGGCCTGAGTGGCCATGTCTCAGCGAAGCAAGTCAATCC |
| 03746ORF-3 | ATAGGCCGCGTTGGCCCCGTCGCCATGCATTCTTCCTTTGAGAAAAATCC |
| 03749ORF-5 | ATAGGCCTGAGTGGCCATGCCACCTGTCGAGAAAACAGGCG |
| 03749ORF-3 | ATAGGCCGCGTTGGCCCCATGGATGTGGCTGAAGAAGGGGTTCG |
| 04580ORF-5 | ATAGGCCTGAGTGGCCATGTCTGAAGCAAATCAGTCGC |
| 04580ORF-3 | ATAGGCCGCGTTGGCCCCAAACCGCCAGCAGCCAAAGTGCG |
| Pmt4ORF-5 | ATAGGCCTGAGTGGCCATGGTCGACGCTACAAAAGCACAGACG |
| Pmt4ORF-3 | ATAGGCCGCGTTGGCCCCCTTTGCAAAGTGGAGCGTCCAGGATGG |
| oDL81 | GTGCCCGACTATGCCGGCGCCTCGACTACTTCAGCCTCGTTGGACAC |
| oDL82 | GTCGTAGGGGTAGGCTGCTCCATTGATGTCGGCTGTATTCTGC |
| oDL124 | ATAGGCCTGAGTGGCCATGGTTCTGTTTCGACCCAAC |
| oDL125 | TATGGCCGCGTTGGCCGCAAGGAGAACCGAGTTGCTCATC |
| oDL171 | TATCCCGGGACCGTCGGCAAGTGCAGGGG |
| oDL204 | ATACCCGGGGCCTCGTTGGACACTTCTCCAAAC |

**Table S4.** Primers used in this study.
